# Cognitive transfer and durability of computerised cognitive training in older adults with mild cognitive impairment: a systematic review and meta-analysis of randomised controlled trials

**DOI:** 10.3389/fnagi.2026.1952783

**Published:** 2026-10-15

**Authors:** Hao Luo, Lin Deng, Lingyun Long, Wenjia Chen, Jiayi Yao, Yong Liu

**Affiliations:** 1 Department of Physical Education, Chongqing Electric Power College, Chongqing, China; 2 College of Education, Soonchunhyang University, Asan, Republic of Korea; 3 School of Physical Education, China University of Mining and Technology, Xuzhou, China

**Keywords:** cognitive transfer, computerised cognitive training, far transfer, meta-analysis, mild cognitive impairment, near transfer, sustained effects

## Abstract

**Background:**

Computerised cognitive training (CCT) may improve cognition in mild cognitive impairment (MCI), but whether gains transfer beyond trained processes and persist after training cessation remains uncertain. We examined immediate and sustained cognitive near transfer, cognitive far transfer, and functional far transfer.

**Methods:**

Seven databases were searched from inception to 1 July 2026. Randomised controlled trials comparing CCT with eligible controls and reporting transfer outcomes were included. Outcomes within 4 weeks after training were classified as immediate and those at least 12 weeks after cessation as sustained. Multiple eligible outcomes from the same trial were aggregated to one trial-level estimate. Random-effects models used restricted maximum likelihood and modified Knapp–Hartung inference. RoB 2 and GRADE assessed risk of bias and certainty.

**Results:**

Eleven reports from eight independent trials (722 randomised participants) were included; no trial was at overall low risk of bias. Immediate cognitive near transfer was small and imprecise (6 trials, *N* = 376; Hedges g = 0.14, 95% CI − 0.23 to 0.50; very low certainty). Immediate cognitive far transfer was also uncertain (8 trials, *N* = 586; g = 0.23, 95% CI − 0.35 to 0.81; very low certainty) and strongly influenced by Wu et al.; excluding this trial yielded g = 0.00 (95% CI − 0.26 to 0.25). Sustained cognitive near transfer (4 trials, *N* = 287; g = −0.07, 95% CI − 0.31 to 0.18) and sustained cognitive far transfer (5 trials, *N* = 397; g = 0.02, 95% CI − 0.26 to 0.29) were close to the null but based on few trials. Functional far-transfer evidence was low-certainty and limited.

**Conclusion:**

The evidence base is small and methodologically limited, and transfer classification requires reviewer judgement. Current evidence does not establish convincing durable transfer effects beyond trained processes. Larger, rigorous trials with validated functional outcomes and longer follow-up are needed.

**Systematic Review Registration:**

https://www.crd.york.ac.uk/prospero/, identifier CRD420261463612.

## 1 Introduction

Population ageing is continuing to increase the disease burden associated with cognitive impairment and dementia. Mild cognitive impairment (MCI) lies between normal cognitive ageing and dementia and is characterised by objective impairment in one or more cognitive domains, while basic independence in daily living is relatively preserved (Petersen, 2016; Albert et al., 2011; Winblad et al., 2004). MCI is markedly heterogeneous and may be classified into amnestic and non-amnestic, as well as single-domain and multidomain, subtypes. Its clinical course is also variable: some individuals progress to dementia, some remain stable, and others may return to near-normal cognitive functioning (GBD 2019 Dementia Forecasting Collaborators, 2019; Rajan et al., 2021; Cong et al., 2023; Qin et al., 2023). Accordingly, MCI represents both a clinical stage associated with an increased risk of dementia and a potentially modifiable window before substantial functional dependence develops. As no pharmacological treatment currently available can reliably reverse the course of MCI, identifying safe and accessible non-pharmacological interventions is of considerable importance.

Computerised cognitive training (CCT) involves the repeated delivery of structured cognitive exercises through computers, tablet devices, digital platforms, or virtual environments. Training may target memory, attention, executive function, processing speed, and other cognitive domains, and some programmes dynamically adjust task difficulty according to participants’ performance while providing immediate feedback (Hill et al., 2017; Zhang et al., 2019; Chan et al., 2024; Gates et al., 2019). Compared with traditional paper-and-pencil or face-to-face training, CCT offers potential advantages, including standardised delivery, individualised progression, and support for remote implementation (Formica et al., 2024; Sheehy et al., 2022; Kwan et al., 2024; Zou et al., 2024). However, improvements in performance within the training platform may primarily reflect task familiarisation, strategy acquisition, or practice effects and do not necessarily demonstrate broad or durable changes in cognitive ability. The clinical value of CCT therefore depends on whether training gains transfer to independent cognitive tests and real-world functioning and whether these gains are maintained after training cessation (Li et al., 2022; Zhong et al., 2021; Papaioannou et al., 2022; Yang et al., 2025). Cognitive near transfer refers to improvement on independent tests assessing cognitive domains that correspond to the training targets; cognitive far transfer refers to improvement in global cognition or in independent tests of domains that were not directly trained; and functional far transfer encompasses instrumental activities of daily living, everyday cognition, and performance-based real-world tasks (Barnett and Ceci, 2002; Borella et al., 2019; Guye and von Bastian, 2017). These three forms of transfer differ in their clinical significance and should be classified according to the relationship between the specific training content and the outcome measure, rather than solely on the basis of the test name (Zuschnegg et al., 2023; Gao and Liu, 2025). Immediate improvements should also be distinguished from sustained benefits: short-term changes after training may be influenced by practice effects or familiarity with the assessment, whereas effects that persist after training cessation are more indicative of durable intervention benefits.

Previous systematic reviews and meta-analyses have suggested that CCT may improve global cognition, memory, or executive function in individuals with MCI, but the existing evidence has three important limitations (Hill et al., 2017; Zhang et al., 2019; Kim et al., 2022; Chen et al., 2024). First, some reviews have not clearly distinguished direct task learning, cognitive near transfer, cognitive far transfer, and functional far transfer, potentially conflating task-specific improvements with more clinically meaningful transfer effects. Second, post-intervention assessments and outcomes measured at different follow-up intervals have often been combined within the same analysis, making it difficult to differentiate short-term responses from sustained effects. Third, a single trial may generate multiple publications, use several outcome measures, and report multiple time points; unless these data are linked and aggregated at the trial level, the same participant sample may be counted more than once, resulting in duplicated weighting and an overestimation of the available information (Borenstein et al., 2009; Hedges and Olkin, 1985; Viechtbauer, 2010). Moreover, real-world functional outcomes and long-term transfer effects have generally been underreported, and it therefore remains uncertain whether CCT can translate into benefits that are perceptible in patients’ everyday lives.

In light of these limitations, this study aimed to systematically review and quantitatively synthesise evidence from randomised controlled trials examining the immediate and sustained effects of CCT on cognitive near transfer, cognitive far transfer, and functional far transfer in middle-aged and older adults with MCI. Secondary objectives were to assess the robustness of the principal findings to individual studies, risk of bias, and assumptions regarding within-trial correlations, and to summarise training adherence, participant withdrawal, and adverse events. In doing so, this study sought to determine whether the benefits of CCT extend beyond the trained tasks to independent cognitive outcomes and real-world functioning and whether they are maintained after training cessation.

## 2 Materials and methods

### 2.1 Protocol registration and search strategy

This systematic review and meta-analysis was conducted and reported in accordance with the PRISMA 2020 statement, and the search process was reported with reference to the PRISMA-S guideline (Page et al., 2020; Rethlefsen et al., 2021). This review was registered in PROSPERO (CRD420261463612). The core methodological framework was prespecified before study screening and data extraction, including the research question, PICOS eligibility criteria, target population, intervention definition, comparator categories, and the intention to evaluate cognitive and functional transfer outcomes separately. Some operational procedures, including transfer classification rules, immediate versus sustained time-window definitions, within-trial outcome aggregation procedures, and sensitivity-analysis specifications, were further clarified before quantitative synthesis to ensure consistent application across heterogeneous eligible trials. These methodological clarifications were applied uniformly across included studies and were not modified according to observed effect sizes or pooled results.

PubMed, Web of Science Core Collection, Embase, the Cochrane Library, Scopus, Wiley Online Library, and CINAHL were systematically searched from database inception to 1 July 2026. Search strategies combined controlled vocabulary with free-text terms in titles and abstracts and were structured around three core concepts: mild cognitive impairment, including MCI and its common subtypes; computerised cognitive training, including computer-based, digital platform-based, mobile device-based, serious game-based, and virtual reality-based cognitive training; and randomised controlled trials. Search terms were adapted to the indexing systems, field structures, and syntax of each database. The search was restricted to formally published studies in English. In addition, the reference lists of included studies and relevant systematic reviews were manually screened, and forward citation searches were conducted in Web of Science and PubMed to identify potentially eligible studies and subsequent reports from the same trial. Full search strategies, search dates, and the number of records retrieved from each database are provided in Supplementary Table S1.

### 2.2 Inclusion and exclusion criteria

Eligibility criteria were defined according to the PICOS framework. (1) Participants (P): middle-aged or older adults diagnosed with mild cognitive impairment (MCI) according to recognised clinical criteria or an equivalent diagnostic procedure, with a study sample mean or median age of at least 60 years in principle. Amnestic, non-amnestic, single-domain, and multidomain MCI were all eligible. Studies in which cognitive impairment was defined solely using Mini-Mental State Examination (MMSE) or Montreal Cognitive Assessment (MoCA) screening cut-offs without a clear diagnosis were excluded. (2) Intervention (I): structured CCT delivered repeatedly through computers, tablet devices, smartphones, digital platforms, or virtual environments, with the primary aim of training one or more cognitive domains. Eligible interventions included adaptive or fixed-difficulty training, single-domain or multidomain training, centre-based supervised training, remotely delivered home-based training, hybrid delivery, serious games, and virtual reality-based training. Multicomponent trials were included only when the independent or incremental effect of CCT could be identified. (3) Comparator (C): wait-list control, no intervention, usual care, health education, attention control, non-adaptive computer activities, active cognitive control, or sham CCT. (4) Outcomes (O): at least one outcome reflecting cognitive near transfer, cognitive far transfer, or functional far transfer. Performance within the training platform was not included in the primary transfer models. Outcomes assessed within 4 weeks after training completion were classified as immediate effects; outcomes assessed >4 but <12 weeks after training cessation were classified as early-maintenance outcomes and retained for supportive description but were not included in the primary immediate or sustained models; outcomes assessed at least 12 weeks after training cessation were classified as sustained effects. (5) Study design (S): formally published randomised controlled trials, including parallel-group, multi-arm, factorial, and pilot trials meeting randomisation requirements.

Exclusion criteria were as follows: (1) quasi-randomised, non-randomised, single-group pre–post, or observational studies; (2) populations that did not meet the prespecified MCI diagnostic or age criteria, or mixed samples in which data for participants with MCI could not be extracted separately; (3) interventions that were not centred on structured CCT, or for which the effect of CCT could not be distinguished from that of other intervention components; (4) studies reporting only performance on trained tasks, neuroimaging outcomes, biomarkers, quality of life, or other outcomes outside the prespecified transfer constructs; (5) reviews, meta-analyses, protocols, conference abstracts, commentaries, editorials, letters, or case reports; (6) duplicate publications from the same trial that provided no additional outcomes or follow-up data; (7) studies with insufficient outcome data to calculate an effect size when the required information remained unavailable after examination of Supplementary materials, trial registration records, or contact with the study authors; and (8) studies that were not published in English or were not formally published.

### 2.3 Study selection, data extraction, and transfer mapping

After deduplication, two reviewers independently screened titles, abstracts, and full texts. Disagreements were resolved through discussion, with arbitration by a third reviewer when necessary. Available reasons for exclusion at the full-text stage were used to generate the PRISMA flow diagram and are summarised in the Supplementary material. A complete citation-level full-text exclusion log with prospectively archived individual screening decisions was not available from the initial screening stage. Protocols, primary outcome reports, secondary outcome reports, and follow-up publications arising from the same randomised sample were linked and assigned a common Trial_ID. The principal unit of analysis was the independent randomised trial rather than the number of articles, reports, or outcome measures.

A prespecified and pilot-tested data extraction form was used to collect information on study design, participant characteristics, intervention and comparator conditions, outcome measures, data required for effect-size calculation, adherence, withdrawals, and safety. One reviewer completed the initial extraction, and a second reviewer independently verified the study characteristics, sample sizes, outcome data, effect directions, transfer classifications, and time windows. Discrepancies were resolved by consulting the original report, Supplementary materials, study protocol, trial registration record, or other reports from the same trial. For each model, the sample size was based on the number of participants actually included in the relevant outcome analysis rather than the total number randomised. A machine-readable extraction sheet containing the study characteristics, outcome-level data, effect sources, transformations, transfer codes, and final trial-level estimates is provided in the Supplementary material to facilitate independent verification.

Effect estimates consistent with the effect of assignment to intervention were prioritised, including intention-to-treat estimates or baseline-adjusted between-group estimates. When these were unavailable, appropriate model-based estimates, post-intervention endpoint data, or change scores were used. The choice of effect source and outcome was not based on the direction of the result or its statistical significance. When multiple eligible measures were reported within the same transfer construct and time window in a single trial, they were first aggregated within the trial to generate one trial-level effect estimate, ensuring that each Trial_ID contributed only once to each primary model.

Using a prespecified coding manual, two reviewers independently classified the trained domain, outcome domain, and type of transfer without reference to the effect size, confidence interval, or statistical significance. Platform-internal tasks or substantively equivalent versions were coded as direct task learning and excluded from the transfer models; an independent test of an explicitly trained cognitive domain was coded as cognitive near transfer; global cognition or an independent test of an untrained domain was coded as cognitive far transfer; and instrumental activities of daily living, everyday cognition, or performance-based real-world tasks were coded as functional far transfer. The same outcome measure could therefore be assigned to different transfer categories depending on the specific content of the training intervention. Disagreements were resolved through discussion or adjudication by a third reviewer. The full coding manual, decision rules, complete data fields, effect sources, transformation records, and the training-domain–outcome-domain–transfer-type mapping table are provided in the Supplementary material. The original pre-consensus independent coding sheets were not retained in a form that permits valid retrospective reconstruction of an agreement statistic; therefore, Cohen’s κ was not calculated from the final consensus dataset. This limitation is reported explicitly below. Adherence, withdrawals, technical difficulties, and adverse events were considered for pooling only when their definitions, time windows, and denominators were sufficiently comparable; otherwise, they were summarised narratively in a structured manner. The absence of reported adverse events was not interpreted as evidence that no adverse events had occurred.

### 2.4 Risk of bias and certainty of evidence assessment

#### 2.4.1 Risk-of-bias assessment

Two reviewers independently assessed the risk of bias for the effect of assignment to intervention using the Cochrane Risk of Bias 2 (RoB 2) tool (Sterne et al., 2019). The assessment unit was the independent trial. For each Trial_ID, the reports and outcomes contributing to this review were considered together to assign one set of domain-level judgements and one overall judgement. When risk-of-bias features differed across outcomes or time points, the trial-level summary reflected the more conservative judgement relevant to the evidence included in the review. The assessment covered five domains: the randomisation process, deviations from intended interventions, missing outcome data, measurement of the outcome, and selection of the reported result. Each domain and the overall risk of bias were judged as low risk, some concerns, or high risk; no summary score was calculated. In behavioural intervention trials, the absence of blinding of participants or intervention providers was not automatically considered indicative of high risk. Particular attention was given to deviations from intended interventions, the analysis population, missing data, and selective reporting. Disagreements were resolved through discussion, with arbitration by a third reviewer when necessary. All eligible trials were included in the primary analyses, and the robustness of the findings was examined in sensitivity analyses excluding trials judged to be at overall high risk of bias.

#### 2.4.2 Certainty of evidence assessment

The GRADE approach was used to assess the certainty of evidence separately for immediate cognitive near transfer (M1), immediate cognitive far transfer (M2), sustained cognitive near transfer (M3), sustained cognitive far transfer (M4), immediate functional far transfer (M5), and sustained functional far transfer (M6). Evidence from randomised controlled trials initially started at high certainty and was downgraded according to risk of bias, inconsistency, indirectness, imprecision, and bias due to missing evidence. The final certainty of evidence was rated as high, moderate, low, or very low (Balshem et al., 2011). Risk-of-bias downgrading was determined at the body-of-evidence level rather than mechanically from the absence of trials at overall low risk; the likely direction and magnitude of bias and the stability of estimates after exclusion of trials at overall high risk were considered when judging whether risk of bias was serious.

Imprecision was assessed by considering the number of trials, outcome-specific sample size, the range of effects encompassed by the 95% CI, the prediction interval, and the overall information size. A Hedges g of 0.20 was used only as a descriptive reference for a small effect and was not treated as a minimum clinically important difference validated in populations with MCI. Each transfer construct and time window was rated separately. For outcomes that did not meet the prespecified threshold for pooling, certainty was assessed on the basis of the individual study findings and the overall size of the evidence base, without forcing a pooled estimate. Detailed GRADE judgements and reasons for downgrading are presented in the summary-of-findings table and Supplementary material S10.

#### 2.4.3 Data analysis

Because the included studies used different cognitive and functional measures, continuous outcomes were converted to the small-sample-corrected standardised mean difference, Hedges g, with corresponding 95% confidence intervals. Effect directions were harmonised so that positive values favoured CCT. For measures in which lower scores indicated improvement, the direction of the effect was reversed. Intention-to-treat or baseline-adjusted between-group estimates were prioritised. When these were unavailable, post-intervention endpoint or change-score data were used, and effect sizes were calculated using the number of participants actually included in the relevant outcome analysis.

When a trial reported multiple eligible outcomes within the same transfer construct and time window, these outcomes were first aggregated within the trial to produce a single trial-level effect estimate, ensuring that each Trial_ID contributed only once to each model. When the covariance could not be obtained directly, a working correlation of ρ = 0.50 between eligible outcomes was assumed for the primary within-trial aggregation. Prespecified sensitivity analyses repeated the complete within-trial aggregation using ρ = 0.30 and ρ = 0.70, while retaining the same trial-level effect selection, random-effects model using restricted maximum likelihood, and modified Knapp–Hartung inference. The aggregation formulae, variance calculations, and data-transformation procedures are described in the Supplementary material.

Separate models were constructed for immediate cognitive near transfer (M1), immediate cognitive far transfer (M2), sustained cognitive near transfer (M3), sustained cognitive far transfer (M4), immediate functional far transfer (M5), and sustained functional far transfer (M6). Transfer constructs and time windows were not combined. M1–M4 were designated as co-primary estimands, whereas M5 and M6 were key secondary estimands. The primary analyses used inverse-variance random-effects models, with between-study variance estimated using restricted maximum likelihood and small-sample inference based on the modified Knapp–Hartung method (Hedges and Olkin, 1985; Knapp and Hartung, 2003; Röver et al., 2015; IntHout et al., 2016). A pooled effect was calculated only when at least three independent trials provided conceptually comparable data; otherwise, the findings of individual studies were reported separately. For each model, Hedges g, the 95% confidence interval, τ^2^, I^2^, and, where applicable, the 95% prediction interval were reported.

Sensitivity analyses included leave-one-out analyses, exclusion of outcomes judged to be at overall high risk of bias according to RoB 2, and re-estimation under alternative assumptions regarding within-trial correlations. Additional influence diagnostics included studentised residuals, leverage (hat) values, Cook’s distance, DFBETAS, and Baujat diagnostics where estimable; influential studies were not excluded solely because of their effect magnitude. When fewer than three trials remained after a sensitivity analysis, a pooled effect was not recalculated. Training dose and comparator type were prespecified as potential effect modifiers. Confirmatory subgroup analyses or univariable meta-regression were prespecified to require at least 10 independent trials, with each subgroup containing at least four trials in principle. As none of the models met this threshold, no confirmatory dose–response meta-regression, comparator-type subgroup analysis, or other confirmatory moderator analysis was conducted. Trial weights were not rescaled according to intervention duration, delivered training time, or comparator category because these variables are potential effect modifiers rather than measures of the sampling precision of an effect estimate. *Post hoc* stratification by mode of training delivery was undertaken solely for hypothesis generation. Funnel plots and Egger’s regression were planned only for models containing at least 10 trials; therefore, no formal assessment of funnel-plot asymmetry was performed.

Training completion, withdrawal, and adverse events were pooled only when their definitions, time windows, and denominators were sufficiently comparable; otherwise, they were summarised narratively in a structured manner. The absence of reported adverse events was not interpreted as evidence that no adverse events had occurred. All analyses were conducted in R version 4.5.2. The complete statistical code, package versions, rules for handling special trial designs, and records of deviations from the analysis plan are provided in the Supplementary material.

## 3 Results

### 3.1 Study selection

A total of 5,050 records were identified through searches of PubMed, Web of Science Core Collection, Embase, the Cochrane Library, Scopus, Wiley Online Library, and CINAHL. After removal of 2,123 duplicate records, 2,927 records underwent title and abstract screening, of which 2,805 were excluded. Full texts were sought for 122 reports; five could not be retrieved, leaving 117 full-text reports for eligibility assessment. Of the 106 full-text reports excluded, 24 were non-randomised studies, 28 had ineligible populations, 34 had ineligible interventions, and 20 did not report an eligible prespecified cognitive or functional transfer outcome. Backward and forward citation searching identified no additional unique records. The complete study-selection process is shown in Figure 1, and the available full-text exclusion records and reasons for exclusion are summarised in the Supplementary material.

**Figure 1:**
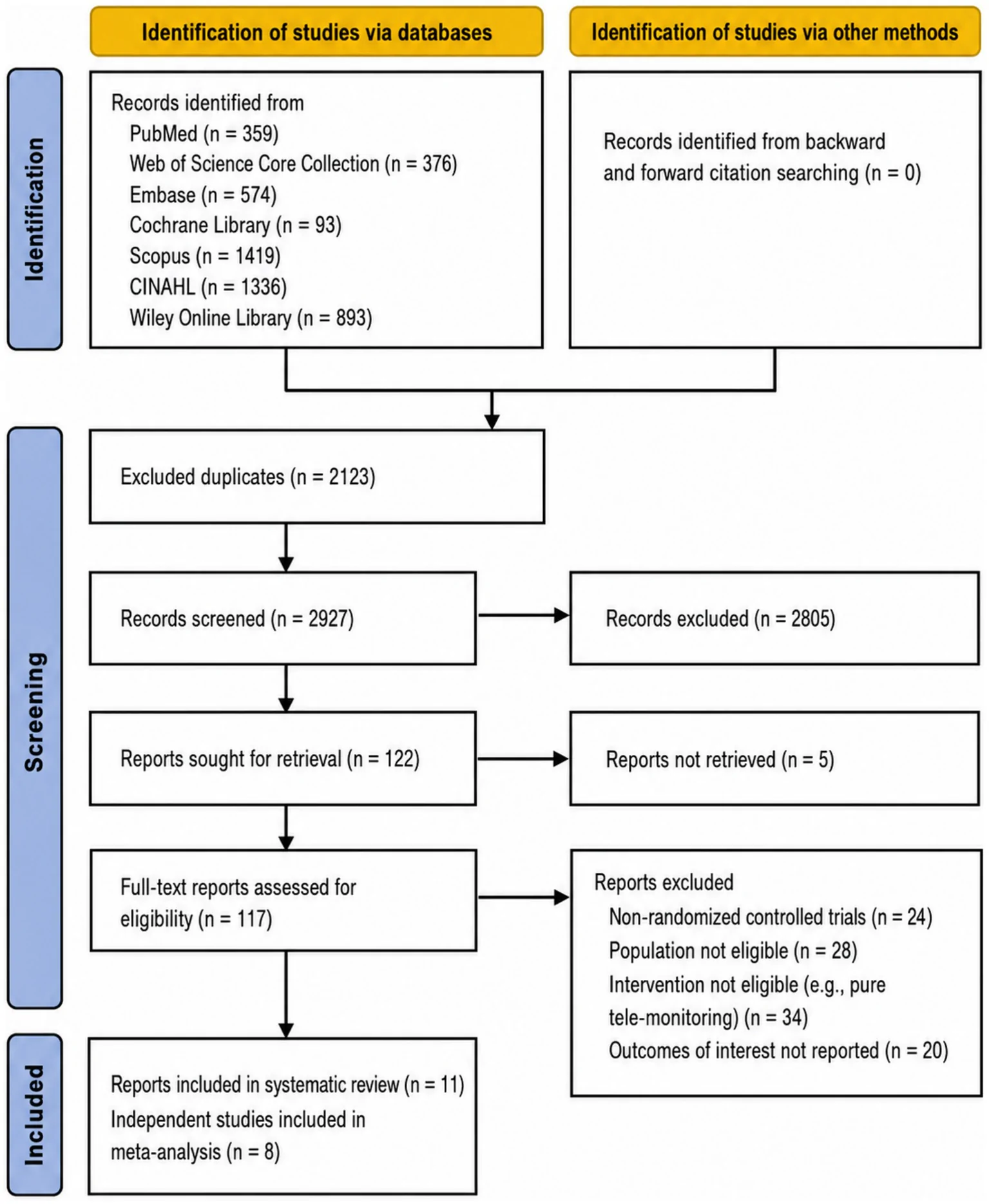
PRISMA 2020 flow diagram of study selection.

Eleven reports from eight independent randomised controlled trials were included. Fiatarone Singh et al. and Broadhouse et al. reported the SMART trial; Dowell-Esquivel et al. and Harvey et al. reported the same trial; and Montero-Odasso et al. and Pieruccini-Faria et al. reported the SYNERGIC trial. Reports from the same trial were linked by Trial_ID and counted once. The relatively small number of independent trials reflected the deliberately narrow estimand and eligibility criteria rather than restriction of the search: eligible studies required a clinically defined MCI population, an isolatable CCT effect, an independent outcome that could be mapped to the prespecified transfer framework, and an assessment assignable to a prespecified time window. Potentially relevant randomised trials identified in previous reviews were re-evaluated against the same criteria rather than automatically inherited. All eight trials contributed to at least one analysis.

### 3.2 Characteristics of the included studies

The 11 included reports represented eight independent randomised controlled trials involving a total of 722 randomised participants. The trials were published between 2014 and 2025 and were conducted in the United States, Australia, Israel, Singapore, Italy, Canada, and China. Randomised sample sizes ranged from 22 to 175 participants per trial. Most studies used individually randomised parallel-group designs, although multi-arm and factorial designs were also represented. In some trials, CCT was compared with sham training against a background of a common exercise intervention, thereby estimating the incremental effect of CCT. All studies enrolled individuals with MCI, although they differed in age range, diagnostic criteria, baseline cognitive status, and the distribution of MCI subtypes. Study populations included individuals with amnestic, non-amnestic, single-domain, and multidomain MCI. Detailed characteristics of the included studies are presented in Table 1.

**Table 1:** Characteristics of the included studies.

Trial (linked reports)	Country/setting	Participants	Sample/age	CCT/comparator	Dose; outcomes; follow-up
Duff et al. (2022)	USA; home-based	Amnestic MCI; ≥65 y	R 73/73; A 55/58 post, 43/51 at 1 y; age 74.9/74.9	Adaptive BrainHQ vs. non-adaptive games	45 min, 4–5/wk., 12–16 wk.; RBANS, ADCS-ADL-MCI; post, 1 y
Fiatarone Singh et al. (2014) and Broadhouse et al. (2020)	Australia; centre-based	Petersen MCI; ≥55 y	*n* = 100 (24/22/27/27); age 70.1	COGPACK and/or PRT vs. sham CCT	60–100 min, 2–3/wk., 6 mo; cognition, Bayer-ADL; 6, 18 mo
Givon Schaham et al. (2024)	Israel; community/home	MCI; MoCA 19–25; >65 y	R 30/31; A 25/25 post, 17/14 at 6 mo; age 74.5/74.0	Tablet TECH vs. usual OT/board games	30–60 min, 3–5/wk., 5 wk.; MoCA, WebNeuro; post, 6 mo
Dowell-Esquivel et al. (2024) and Harvey et al. (2025)	USA; remote/home	Jak–Bondi MCI; MoCA ≥18; ≥60 y	R 54/55; A 47/45; age 71.7	BrainHQ + FUNSAT vs. FUNSAT	2/wk., ≤12 wk.; cognition, performance-based function; post, 30 d
Kua et al. (2025)	Singapore; clinic	Petersen MCI; MMSE ≥23; 65–85 y	R 11/11; A 11/9	MindGym VR vs. usual care + cognitive stimulation	60 min, 1/wk., 10 wk.; MoCA, FAB, RBANS; 10 wk
Manenti et al. (2020)	Italy; centre/home	Petersen MCI; MMSE 24–30; CDR 0.5; >65 y	*n* = 49 (18/14/17); age 75.3/76.3/78.1	VRRS ± home training vs. usual rehabilitation	60 min, 3/wk.; 4 wk. centre + 12 wk. home; cognition, ADL/IADL; 1, 4, 7 mo
Montero-Odasso et al. (2023) and Pieruccini-Faria et al. (2025)	Canada; multicentre	MCI; objective impairment, preserved function; 60–85 y	*n* = 175 (34/35/37/35/34); age 72.4–73.8	Adaptive CCT + exercise vs. sham CCT + exercise (vitamin D factorial)	90 min, 3/wk., 20 wk.; ADAS-Cog, domains, IADL; post, 6, 12 mo
Wu et al. (2023)	China; hospital	Petersen MCI; MoCA ≤25; GDS 2–3; 50–85 y	R 30/30; A 29/28; age 67.7/65.5	11-module adaptive CCT vs. health education	60 min, 3/wk., 8 wk.; MoCA, DSST, Stroop, CAVLT; post ≤1 wk

Reports originating from the same randomised trial were grouped together and assigned a common Trial_ID. Each trial was counted once in the meta-analysis.

The CCT interventions were heterogeneous and included single-domain or multidomain training, as well as adaptive or fixed-difficulty programmes. Training primarily targeted memory, attention, executive function, processing speed, and multidomain cognitive abilities. Intervention duration ranged from approximately 3 weeks to 6 months, with training delivered 1–5 times per week for 30–100 min per session. Delivery formats included supervised centre- or outpatient-based training, remotely delivered home-based training, and hybrid centre–home approaches. Comparator conditions included usual care, health education, general cognitive stimulation, non-adaptive computer activities, sham CCT, and other active interventions. Consequently, some studies estimated the incremental effect of CCT relative to an active comparator rather than its absolute effect relative to no intervention.

Study outcomes included global cognition, domain-specific neuropsychological tests, instrumental activities of daily living, everyday cognition, and performance-based measures of real-world functioning. All eight trials contributed data to at least one prespecified transfer model, although the amount of evidence varied across transfer constructs and time windows. Immediate cognitive outcomes were reported more frequently, whereas sustained cognitive outcomes and functional far-transfer outcomes were less commonly available. Follow-up periods extended from the end of the intervention to 18 months. The 30-day assessment from the Dowell-Esquivel et al. (2024) trial was classified within the early-maintenance window and was not included in the sustained-effect models requiring at least 12 weeks after training cessation.

### 3.3 Risk of bias

According to the Cochrane RoB 2 tool, the Dowell-Esquivel et al. (2024) trial, Wu et al., and Givon Schaham et al. were judged to be at overall high risk of bias, while the remaining five trials raised some concerns; no trial was judged to be at overall low risk of bias (Dowell-Esquivel et al., 2024; Wu et al., 2023; Givon Schaham et al., 2024). Detailed trial-level judgements for each study and domain are presented in Figures 2A,B.

**Figure 2:**
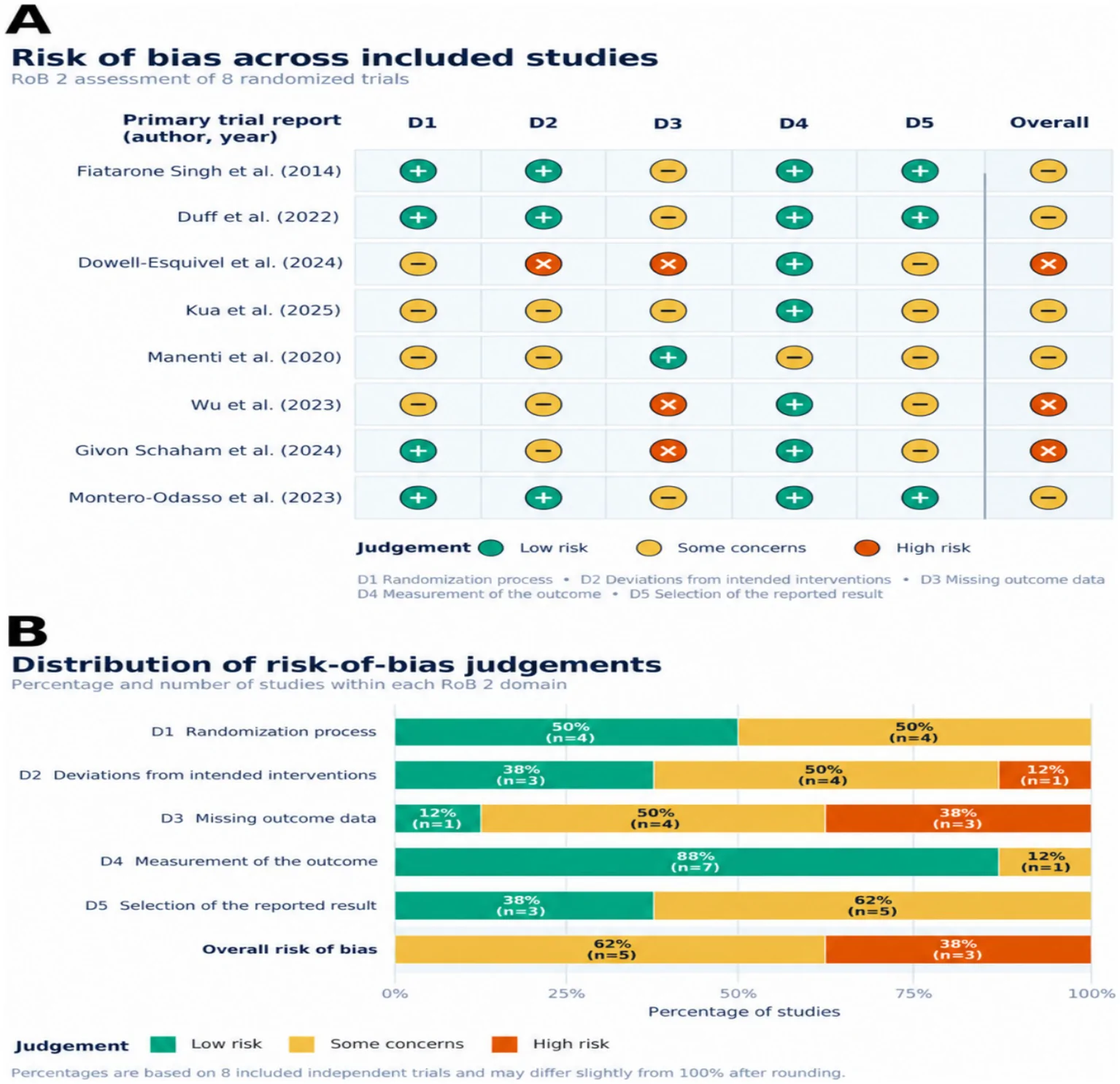
Risk of bias among the included trials. **(A)** Trial-level RoB 2 judgements; **(B)** distribution of judgements across RoB 2 domains and overall risk of bias. D1, bias arising from the randomisation process; D2, bias due to deviations from intended interventions; D3, bias due to missing outcome data; D4, bias in measurement of the outcome; D5, bias in selection of the reported result.

Missing outcome data were the principal source of bias. In domain D3, one trial was judged to be at low risk, four raised some concerns, and three were judged to be at high risk. For the randomisation process, four trials were judged to be at low risk and four raised some concerns, mainly because of insufficient reporting of random sequence generation, allocation concealment, or baseline comparability. In the domain concerning deviations from intended interventions, three trials were judged to be at low risk, four raised some concerns, and one was judged to be at high risk. For selection of the reported result, three trials were judged to be at low risk and five raised some concerns. Risk related to outcome measurement was comparatively low, with seven trials judged to be at low risk and one raising some concerns.

Overall high risk of bias was driven primarily by missing data and analyses that were not fully consistent with estimating the effect of assignment to intervention. Wu et al. analysed only the 50 participants who completed the cognitive and neuroimaging assessments (Wu et al., 2023). In the trial by Givon Schaham et al., only 31 of the 61 randomised participants completed the 6-month follow-up, and missing data were handled using the last observation carried forward method (Givon Schaham et al., 2024). The Dowell-Esquivel et al. (2024) trial showed discrepancies between the analysed and randomised samples, together with insufficient information on adherence and deviations from the intended intervention. These judgements informed the prespecified sensitivity analyses excluding trials at overall high risk of bias (Dowell-Esquivel et al., 2024; Harvey et al., 2025).

### 3.4 Cognitive transfer effects

All eight independent randomised controlled trials contributed data to at least one prespecified model. Effect directions were harmonised so that positive values favoured CCT. Cognitive near transfer and cognitive far transfer were modelled separately, and immediate effects assessed within 4 weeks after training completion were analysed separately from sustained effects assessed at least 12 weeks after training cessation.

#### 3.4.1 Immediate cognitive near transfer

Six trials involving 376 participants were included in the immediate cognitive near-transfer model. The pooled effect was small, and the 95% confidence interval crossed the line of no effect (Hedges g = 0.14, 95% CI − 0.23 to 0.50, *p* = 0.373). Between-study heterogeneity was high (Q = 18.47, df = 5, *p* = 0.002; I^2^ = 72.9%; τ^2^ = 0.081), and the 95% prediction interval ranged from −0.74 to 1.02, indicating that the true effects in future similar studies may vary substantially (Figure 3a).

**Figure 3:**
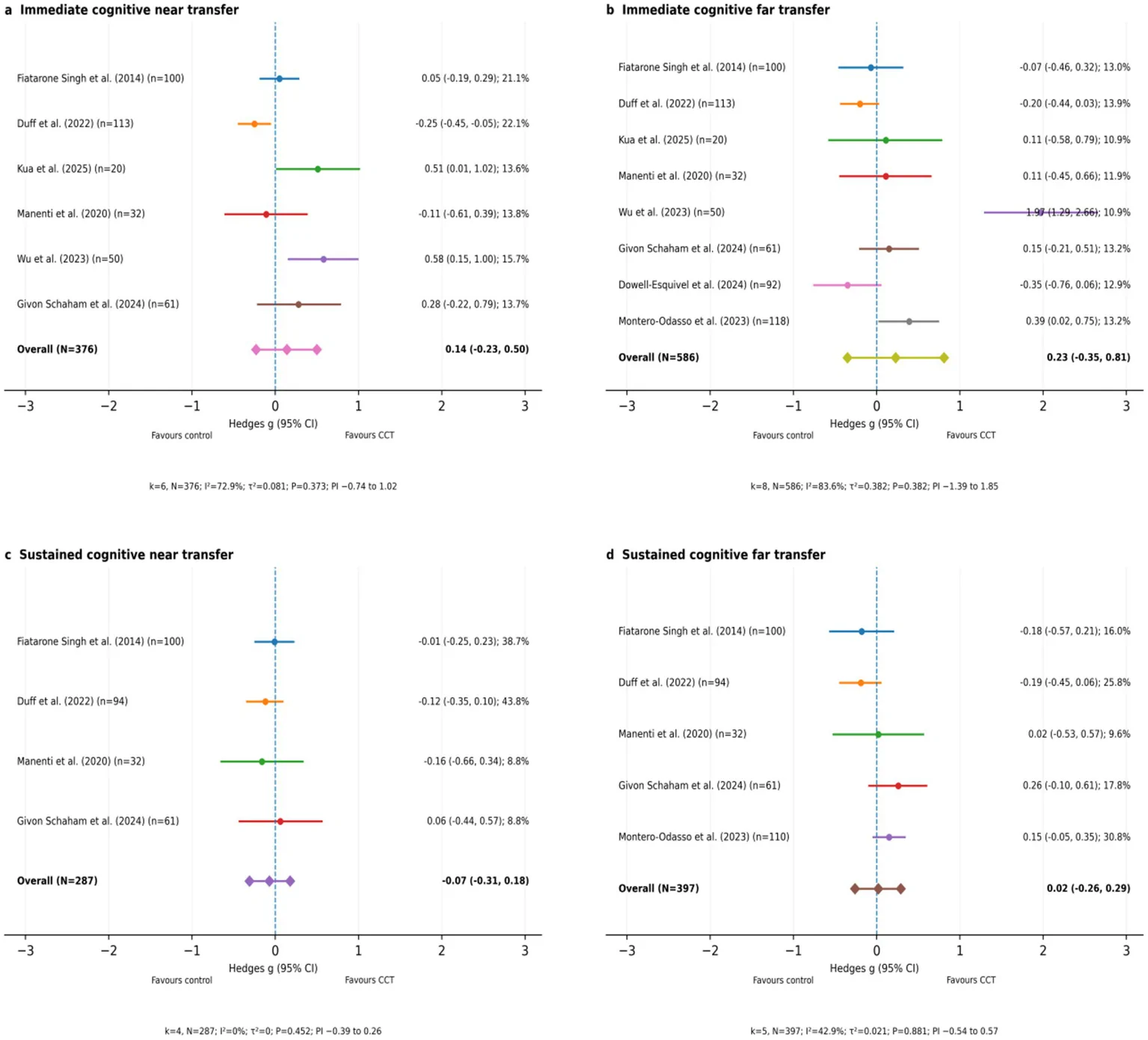
Cognitive transfer effects: **(a)** immediate cognitive near transfer (M1); **(b)** immediate cognitive far transfer (M2); **(c)** sustained cognitive near transfer (M3); and **(d)** sustained cognitive far transfer (M4). Positive values favour CCT.

#### 3.4.2 Immediate cognitive far transfer

Eight trials involving 586 participants were included in the immediate cognitive far-transfer model. The pooled point estimate favoured CCT, but the 95% confidence interval was wide and crossed the line of no effect (Hedges g = 0.23, 95% CI − 0.35 to 0.81, *p* = 0.382). Between-study heterogeneity was high (Q = 42.79, df = 7, *p* < 0.001; I^2^ = 83.6%; τ^2^ = 0.382), and the 95% prediction interval ranged from −1.39 to 1.85. The effect reported by Wu et al. was substantially larger than those of the other studies (Hedges g = 1.97, 95% CI 1.29 to 2.66), whereas the effects in the remaining trials were generally close to the null or inconsistent in direction (Figure 3b).

#### 3.4.3 Sustained cognitive near transfer

Four trials involving 287 participants reported cognitive near-transfer outcomes at least 12 weeks after training cessation. The pooled effect was close to the null (Hedges g = −0.07, 95% CI − 0.31 to 0.18, *p* = 0.452), and no statistically detectable between-study heterogeneity was observed (Q = 0.85, df = 3, *p* = 0.838; I^2^ = 0%; τ^2^ = 0). The 95% prediction interval ranged from −0.39 to 0.26; however, its stability was limited because only four trials were included (Figure 3c).

#### 3.4.4 Sustained cognitive far transfer

Five trials involving 397 participants were included in the sustained cognitive far-transfer model. The pooled effect was close to the null (Hedges g = 0.02, 95% CI − 0.26 to 0.29, *p* = 0.881). Between-study heterogeneity was lower than that observed in the immediate cognitive far-transfer model (Q = 7.01, df = 4, *p* = 0.136; I^2^ = 42.9%; τ^2^ = 0.021), and the 95% prediction interval ranged from −0.54 to 0.57 (Figure 3d).

### 3.5 Functional far-transfer effects

Functional far transfer encompassed instrumental activities of daily living, everyday cognition, and performance-based measures of real-world functioning. Immediate effects assessed within 4 weeks after training completion and sustained effects assessed at least 12 weeks after training cessation were analysed separately (Dowell-Esquivel et al., 2024; Fiatarone Singh et al., 2014; Duff et al., 2022; Pieruccini-Faria et al., 2025). A pooled effect was calculated only when at least three independent trials provided conceptually comparable data.

#### 3.5.1 Immediate functional far transfer

Three trials involving 305 participants were included in the immediate functional far-transfer model. The pooled effect slightly favoured the control condition, but the 95% confidence interval crossed the line of no effect (Hedges g = −0.16, 95% CI − 0.66 to 0.33, *p* = 0.296). No statistically detectable between-study heterogeneity was observed (Q = 1.06, df = 2, *p* = 0.588; I^2^ = 0%; τ^2^ = 0). The 95% prediction interval ranged from −1.62 to 1.30; however, the stability of this estimate was limited because only three trials were included (Figure 4a).

**Figure 4:**
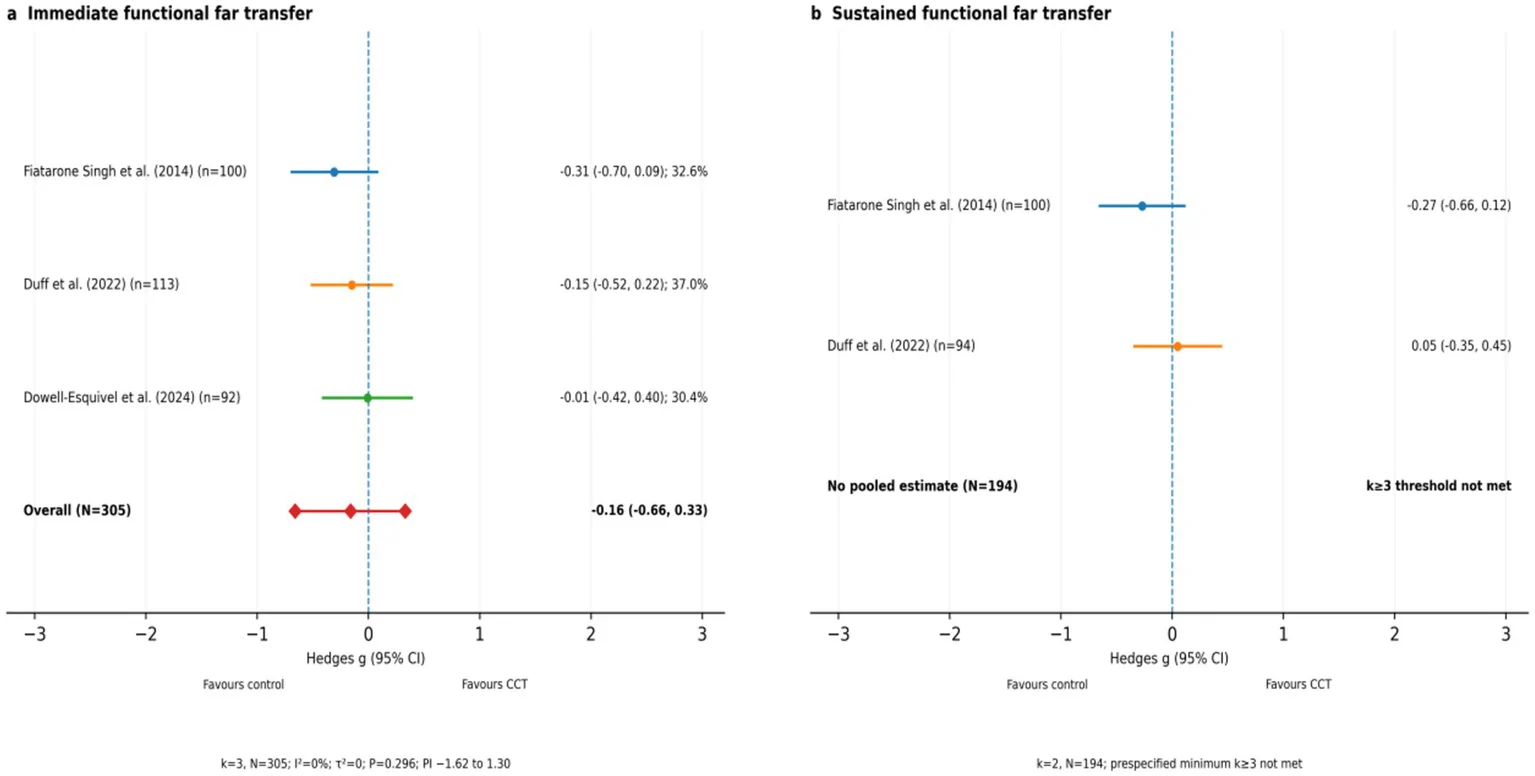
Functional far-transfer effects: **(a)** immediate functional far transfer (M5); **(b)** sustained functional far transfer (M6). No pooled estimate was calculated for M6 because the prespecified minimum of three independent trials was not met.

#### 3.5.2 Sustained functional far transfer

Only two trials involving 194 participants reported functional far-transfer outcomes at least 12 weeks after training cessation. The effect reported by Fiatarone Singh et al. slightly favoured the control group (Hedges g = −0.27, 95% CI − 0.66 to 0.12), whereas the effect reported by Duff et al. was close to the null (Hedges g = 0.05, 95% CI − 0.35 to 0.45).

Because the prespecified minimum threshold for pooling was not met (k ≥ 3), no pooled estimate was calculated for sustained functional far transfer. The findings from the two studies are presented separately in the forest plot (Figure 4b).

### 3.6 Sensitivity analyses

Leave-one-out analysis showed that, after exclusion of Wu et al., the pooled effect for immediate cognitive far transfer decreased from 0.23 to approximately zero (Hedges g = 0.00, 95% CI − 0.26 to 0.25), indicating that this trial had a substantial influence on the overall point estimate (Wu et al., 2023). Additional influence diagnostics likewise identified Wu et al. as the most influential trial in M2: it was the only study with an absolute studentised residual exceeding 2 and it had the largest Cook’s distance. No study was removed from the primary analysis solely because it was influential. The remaining leave-one-out analyses did not materially alter the principal conclusions. Complete influence diagnostics are presented in the Supplementary material.

After excluding the Dowell-Esquivel et al. (2024) trial and the trials by Wu et al. and Givon Schaham et al., which were judged to be at overall high risk of bias, the effect for immediate cognitive near transfer decreased to Hedges g = 0.00 (95% CI − 0.48 to 0.49; 4 trials, *N* = 265), while the effect for immediate cognitive far transfer decreased to 0.04 (95% CI − 0.32 to 0.39; 5 trials, *N* = 383). For the latter outcome, I^2^ decreased from 83.6 to 47.0%. The sustained cognitive near-transfer estimate remained close to the null after exclusion of high-risk trials (Hedges g = −0.08, 95% CI − 0.42 to 0.26; 3 trials, *N* = 226), as did the sustained cognitive far-transfer estimate (Hedges g = −0.04, 95% CI − 0.38 to 0.31; 4 trials, *N* = 336). These restricted analyses remained imprecise and were not interpreted as demonstrating absence of a durable effect. After exclusion of high-risk trials, only two trials remained in the immediate functional far-transfer analysis; therefore, no pooled effect was recalculated.

### 3.7 Exploratory stratified analyses and small-study effects

Each model included only 2–8 independent trials and therefore did not meet the prespecified threshold for formal subgroup analysis or meta-regression. Training dose and comparator type were prespecified potential effect modifiers, but the evidence base was too sparse for reliable dose meta-regression or comparator-specific interaction testing, and several comparator categories contained only one or two trials. Study weights were therefore retained on the basis of inverse-variance precision rather than rescaled according to intervention exposure or comparator intensity. After substantial heterogeneity was observed in the immediate cognitive transfer models, *post hoc* exploratory stratification was conducted according to mode of training delivery. Point estimates were relatively larger in the centre-based or therapist-supervised training subgroup, although the confidence intervals were wide, whereas estimates in the home-based, remote, or hybrid training subgroup were close to zero (Dowell-Esquivel et al., 2024; Duff et al., 2022; Kua et al., 2025; Manenti et al., 2020). Because no adequately powered formal interaction test was performed, these findings were considered hypothesis-generating only and cannot be used to determine whether one mode of delivery is superior to another (Supplementary Figures S3A,B).

All models included fewer than 10 trials; therefore, no formal assessment of funnel-plot asymmetry or Egger’s regression was conducted (Egger et al., 1997; Sterne et al., 2011). The absence of these analyses does not exclude small-study effects, publication bias, or other forms of bias due to missing evidence.

### 3.8 Adherence, withdrawals, and adverse events

All eight trials reported the planned training protocol, but the definitions and reporting of actual training dose, training completion, and adherence were inconsistent. Participant retention also varied across studies and follow-up periods, with longer-term follow-up generally accompanied by reductions in sample size. Because the definitions, time windows, and denominators used for training completion and withdrawal were not comparable, training completion and withdrawal rates were not quantitatively pooled.

Reporting of adverse events and technical feasibility was generally limited. Most studies did not use consistent denominators when reporting CCT-related discomfort, equipment or network failures, serious adverse events, or the relationship of such events to the intervention. Accordingly, adverse-event proportions were not pooled. The absence of reported adverse events was not interpreted as evidence that no adverse events had occurred.

### 3.9 Progression to dementia and long-term cognitive worsening

Comparable diagnostic outcomes on conversion from MCI to dementia were generally not reported. Duff et al. provided the only directly related long-term indicator: over 1 year, six participants developed functional difficulties suggestive of dementia, with no clear difference between the experimental and control groups (χ^2^[1] = 0.43, *p* = 0.41); the trial authors explicitly noted that the 1-year follow-up was insufficient to determine whether CCT altered conversion from MCI to dementia (Duff et al., 2022). Other included trials did not provide comparable randomised-group data on clinically diagnosed conversion to dementia. Consequently, no quantitative synthesis or inference regarding dementia prevention or disease-modifying effects was undertaken.

### 3.10 Certainty of evidence

The GRADE assessments are presented in the summary-of-findings table. The certainty of evidence for both immediate cognitive near transfer and immediate cognitive far transfer was rated as very low, primarily because of downgrading for risk of bias, inconsistency, and imprecision. The immediate cognitive near-transfer findings were sensitive to trials at high risk of bias, whereas both the pooled effect and heterogeneity for immediate cognitive far transfer were substantially influenced by an individual high-risk trial. In addition, the confidence and prediction intervals were wide.

The certainty of evidence for sustained cognitive near transfer and sustained cognitive far transfer was rated as moderate. No included trial was judged to be at overall low risk of bias; however, GRADE risk-of-bias judgements were made for the body of evidence rather than by automatically downgrading whenever all studies had some concerns. After exclusion of trials at overall high risk of bias, the sustained near-transfer estimate remained Hedges g = −0.08 (95% CI − 0.42 to 0.26; 3 trials, *N* = 226) and the sustained far-transfer estimate remained Hedges g = −0.04 (95% CI − 0.38 to 0.31; 4 trials, *N* = 336). Because these restrictions did not materially alter the direction or magnitude of the pooled estimates, risk of bias was not judged serious enough to warrant an additional downgrade for M3 or M4. Both outcomes were nevertheless downgraded for imprecision because only four to five trials contributed to the primary models, sample sizes were limited, and the confidence intervals remained compatible with modest beneficial and harmful effects. Accordingly, the moderate-certainty rating should be interpreted as moderate confidence in the estimated average effect, not as proof that a durable effect is absent.

The certainty of evidence for both immediate and sustained functional far transfer was rated as low. Immediate functional far transfer was informed by only three trials, with wide confidence and prediction intervals. Sustained functional far transfer was reported in only two trials and did not meet the prespecified threshold for pooling. Both functional outcomes were downgraded primarily because of the limited evidence base and serious imprecision (Table 2).

**Table 2:** Main findings and certainty of evidence for transfer outcomes.

Outcome	No. of trials (participants)	Effect estimate	95% prediction interval	Certainty of evidence	Interpretation
Immediate cognitive near transfer (M1)	6 RCTs (*N* = 376)	Hedges g = 0.14 (95% CI − 0.23 to 0.50)	−0.74 to 1.02	Very low ⨁◯◯◯	The effect was small and highly uncertain and was sensitive to trials at high risk of bias
Immediate cognitive far transfer (M2)	8 RCTs (*N* = 586)	Hedges g = 0.23 (95% CI − 0.35 to 0.81)	−1.39 to 1.85	Very low ⨁◯◯◯	The average effect was imprecise, with substantial between-study heterogeneity and marked influence from an individual trial
Sustained cognitive near transfer (M3)	4 RCTs (*N* = 287)	Hedges g = −0.07 (95% CI − 0.31 to 0.18)	−0.39 to 0.26	Moderate ⨁⨁⨁◯	The pooled estimate was close to the null, but only four trials contributed; modest benefit or harm cannot be excluded, and absence of a durable effect is not established
Sustained cognitive far transfer (M4)	5 RCTs (*N* = 397)	Hedges g = 0.02 (95% CI − 0.26 to 0.29)	−0.54 to 0.57	Moderate ⨁⨁⨁◯	The pooled estimate was close to the null, but only five trials contributed; modest benefit or harm cannot be excluded, and absence of a durable effect is not established
Immediate functional far transfer (M5)	3 RCTs (*N* = 305)	Hedges g = −0.16 (95% CI − 0.66 to 0.33)	−1.62 to 1.30	Low ⨁⨁◯◯	The evidence base was limited, and the presence of an immediate functional benefit remains uncertain
Sustained functional far transfer (M6)	2 RCTs (*N* = 194)	Not pooled; individual effects were −0.27 and 0.05	Not applicable	Low ⨁⨁◯◯	The prespecified threshold for pooling was not met, and sustained functional effects remain uncertain

CCT, computerised cognitive training; CI, confidence interval; GRADE, Grading of Recommendations Assessment, Development and Evaluation; MCI, mild cognitive impairment; RCT, randomised controlled trial. Positive Hedges’ g values favour CCT.

## 4 Discussion

### 4.1 Principal findings

This systematic review included eight independent randomised controlled trials. The findings showed that the immediate cognitive transfer effects of CCT in middle-aged and older adults with MCI were small and unstable, whereas sustained cognitive transfer effects assessed at least 12 weeks after training cessation were generally close to zero. The point estimates for both immediate cognitive near transfer and immediate cognitive far transfer favoured CCT, but the confidence intervals crossed the line of no effect, between-study differences were substantial, and the certainty of evidence for both outcomes was very low. Accordingly, the current findings are insufficient to support the conclusion that CCT produces consistent immediate transfer benefits across different populations, training programmes, and research settings.

The immediate cognitive transfer findings were also sensitive to trials at high risk of bias and to highly influential studies. After trials judged to be at overall high risk of bias were excluded, the pooled effects for both immediate near and far transfer decreased to values close to zero. In particular, the trial by Wu et al. had a marked influence on both the point estimate and heterogeneity for immediate cognitive far transfer. By contrast, the pooled estimates for sustained cognitive near and far transfer remained close to the null after exclusion of high-risk trials. However, only four to five trials contributed to the primary sustained models, no included trial was at overall low risk of bias, and the confidence intervals remained compatible with modest effects. The stability of these estimates should therefore not be interpreted as confirmation that durable effects are absent. Rather, the available evidence does not provide convincing evidence of a durable average cognitive transfer benefit, while modest beneficial or harmful effects remain possible.

Evidence for functional far transfer was even more limited. Immediate functional far transfer was informed by only three trials and was imprecisely estimated, whereas sustained functional far transfer was reported in only two trials and did not meet the prespecified threshold for pooling. The certainty of evidence for both functional outcomes was low. Overall, improvements observed in trained tasks or related cognitive tests cannot be directly interpreted as evidence of benefits in untrained cognitive domains, everyday functioning, or long-term clinical outcomes.

### 4.2 Interpretation of the findings and comparison with previous evidence

CCT typically strengthens specific cognitive operations, response rules, and task strategies through repeated practice, progressive increases in difficulty, and immediate feedback. Its benefits may therefore remain largely confined to tests that closely resemble the trained content (Barnett and Ceci, 2002; Guye and von Bastian, 2017; Kawata et al., 2022; Dai et al., 2024). Even when outcomes are assessed using independent neuropsychological tests, the degree of overlap between the training tasks and outcome measures in stimulus format, cognitive demands, and response requirements may influence the magnitude of transfer. Different tasks within the same cognitive domain are not necessarily equivalent. For example, memory tests may rely to varying degrees on encoding, retrieval, working memory, and attentional control, whereas executive function tests may primarily assess inhibition, switching, or planning (Nicastri et al., 2022; Antonenko et al., 2022; Liu et al., 2023; Ben Izhak et al., 2024). Thus, classification within the same broad cognitive domain does not necessarily imply that the training and outcome measure engage the same cognitive processes, which may partly explain the inconsistent immediate near-transfer effects.

Differences in comparator conditions and training protocols may also have influenced the immediate effects. Some studies used active comparators such as health education, general cognitive stimulation, non-adaptive games, sham CCT, or functional skills training. These activities may themselves produce cognitive engagement, familiarity with the equipment, social contact, and repeated-testing effects (Dowell-Esquivel et al., 2024; Montero-Odasso et al., 2023; Motter et al., 2024; Hwang et al., 2023). Consequently, some studies estimated the incremental effect of CCT relative to another active activity rather than its absolute effect relative to no training. Differences in trained cognitive domains, adaptivity, prescribed and actually completed training dose, supervision, and delivery setting may have further increased variation in effects across studies. Training dose and comparator type were prespecified as potential effect modifiers, but with at most eight independent trials per model the review did not have sufficient information for reliable meta-regression or formal subgroup interaction tests. Reweighting trials by intervention time or comparator category was not used because meta-analytic weights reflect statistical precision, whereas dose and comparator intensity are potential determinants of the underlying effect.

The near-null sustained pooled estimates may partly reflect attenuation after training cessation, but they may also reflect limited statistical power, heterogeneous follow-up, or insensitivity of the available outcome measures. Some immediate improvements may primarily represent task proficiency, strategy learning, or transient cognitive activation and may gradually diminish in the absence of continued practice. Few included studies systematically incorporated maintenance or booster training, and actual completed training dose was reported inconsistently (Manenti et al., 2020; Broadhouse et al., 2020; Booth et al., 2023; He et al., 2022). In addition, MCI is heterogeneous in aetiology, subtype, and clinical trajectory, and small short-term training gains may be insufficient to offset age-related changes, neurodegenerative processes, and comorbid conditions over the longer term. Inconsistent follow-up periods, attrition during long-term follow-up, and limited sensitivity of outcome measures to subtle changes may also have reduced the ability to detect sustained effects (Givon Schaham et al., 2024; Thomas et al., 2022; Kim et al., 2023; Baker et al., 2025).

Previous meta-analyses have generally suggested that CCT may improve global cognition, memory, or working memory in individuals with MCI (Hill et al., 2017; Chan et al., 2024; Jeong et al., 2025; Ye et al., 2024). For example, the systematic review by Hill et al. reported potential benefits across several cognitive domains, and more recent large-scale meta-analyses have also identified favourable effects on global cognition. However, these reviews generally pooled post-intervention outcomes according to the cognitive domain assigned to each measure, with limited differentiation among direct task learning, cognitive near transfer, cognitive far transfer, and functional far transfer on the basis of the specific training targets. Immediate and longer-term follow-up outcomes were also not consistently analysed separately (Zhang et al., 2019; Tortora et al., 2024; Ren et al., 2024; Cai et al., 2024).

The differences between the present review and previous evidence primarily arise from differences in the estimand and unit of analysis. In this study, transfer distance was determined according to the relationship between the training targets and independent outcome measures, and immediate and sustained effects were modelled separately. Multiple reports, outcome measures, and repeated time points from the same trial were first linked and aggregated within the trial. Consequently, the effect estimates in this review may be more conservative than those obtained in previous analyses that pooled global cognition or individual cognitive domains. However, they more directly address whether training benefits extend beyond the practised tasks, persist after training cessation, and translate into real-world functioning.

### 4.3 Heterogeneity and robustness of the findings

The effect reported by Wu et al. in the immediate cognitive far-transfer model was substantially larger than those of the other trials and had a marked influence on the overall point estimate (Wu et al., 2023). Leave-one-out analysis reduced the pooled estimate to approximately zero when this study was omitted, and additional influence diagnostics identified Wu et al. as the most influential trial in M2, including the only absolute studentised residual exceeding 2 and the largest Cook’s distance. Heterogeneity in the immediate cognitive far-transfer model also decreased substantially after all trials at overall high risk of bias were excluded. The point estimate for immediate cognitive near transfer likewise decreased to approximately zero after exclusion of high-risk trials, indicating that the immediate findings were sensitive to study quality and individual-study influence.

However, an influential study should not be excluded solely because it reports a large effect size. The principal concern regarding Wu et al. was that only 50 of the 60 randomised participants were included in the cognitive and neuroimaging analyses. The effect estimate was therefore based primarily on the subgroup with complete data, which may have differed systematically from the original randomised population. The available information was insufficient to determine the direction or magnitude of any resulting bias. Retaining this trial in the primary model and evaluating its influence through leave-one-out analyses and sensitivity analyses excluding high-risk trials was therefore more methodologically cautious than removing it on the basis of the observed results.

Beyond individual influential trials, the studies differed in participant age, MCI diagnostic criteria and subtypes, baseline cognitive status, training content, adaptivity, training dose, mode of delivery, comparator intensity, outcome measures, and follow-up duration. Studies also used different analytical approaches, including baseline-adjusted models, endpoint data, and complete-case analyses, and handled missing data inconsistently. Heterogeneity in the immediate-effect models was therefore more likely to reflect a combination of clinical and methodological factors than any single source. The *post hoc* stratification by mode of training delivery was intended solely to generate hypotheses; it does not demonstrate that delivery mode explained the heterogeneity or support the superiority of one training approach over another.

### 4.4 Clinical and research implications

CCT offers potential advantages, including standardised training content, suitability for repeated delivery, and support for home-based or remote implementation. However, short-term improvements on cognitive tests should not be equated directly with clinical benefit. The included trials did not provide sufficient comparable data to determine whether CCT altered conversion from MCI to dementia or clinically defined long-term cognitive deterioration. One trial reported six participants with functional difficulties suggestive of dementia at 1 year, without a clear between-group difference, but explicitly considered its follow-up too short to assess conversion (Duff et al., 2022). Thus, the present review cannot support inference about disease modification or dementia prevention. On the basis of the transfer outcomes currently available, there is no convincing evidence that CCT consistently produces durable cognitive or real-world functional benefits, but the limited evidence base also does not rule out modest or subgroup-specific durable effects. In clinical practice, CCT may be considered a potential component of an individualised cognitive support programme, but it should not replace comprehensive interventions addressing physical activity, vascular risk-factor management, social participation, sleep, and emotional health.

Home-based and remotely delivered training may reduce barriers related to transport and access to training facilities, but its effectiveness in practice also depends on digital literacy, sensory function, equipment and internet access, caregiver support, and the need for technical supervision. Most trials described the planned training protocol in reasonable detail, but few used standardised methods to report actual training duration, completed dose, adherence thresholds, or technical difficulties. Consequently, the difference between the prescribed dose and the intensity of training actually received remains difficult to determine.

Future trials should use explicit MCI diagnostic criteria and adequately powered multicentre designs, with primary transfer outcomes, assessment time points, and statistical methods prespecified in trial registrations and protocols. In addition to post-intervention assessments, sufficiently long follow-up periods should be incorporated to determine whether maintenance training, booster sessions, or continued access to the training platform improves the durability of effects. Outcome assessment should include both independent cognitive tests and patient-important measures of real-world functioning, particularly instrumental activities of daily living, everyday cognition, and performance-based functional measures, rather than relying solely on trained tasks or global cognitive scales.

Future studies should also standardise the operational definitions of training completion and adherence, use platform-generated records to capture actual training time, task completion, progression in difficulty, and interruptions, and systematically report requirements for technical support, reasons for withdrawal, and adverse events. As the evidence base expands, individual participant data meta-analysis and component or dose–response analyses could be used to examine whether MCI subtype, baseline cognitive status, digital literacy, training content, and actual training dose modify the response to intervention.

### 4.5 Strengths and limitations

The principal strength of this study was the use of a prespecified “transfer construct–time window” framework to evaluate the immediate and sustained effects of cognitive near transfer, cognitive far transfer, and functional far transfer separately. The primary analyses used the independent Trial_ID as the unit of analysis and linked multiple reports, outcome measures, and repeated time points from the same trial before aggregating them within trials, thereby reducing duplicated weighting. The statistical analyses used random-effects models with restricted maximum likelihood estimation and modified Knapp–Hartung small-sample inference, together with prediction intervals, sensitivity analyses, RoB 2, and GRADE to characterise uncertainty in the findings. The number of participants in each model was also based on the sample actually included in the corresponding outcome analysis rather than the total number randomised.

First, the evidence base was limited, with only 2–8 independent trials included in each model; evidence for functional far transfer and sustained outcomes was particularly sparse. The small number of trials reduced the precision of the estimated effects, heterogeneity, and prediction intervals and precluded formal subgroup analyses, meta-regression, and tests for small-study effects. The methodological quality of the included studies was also suboptimal. No trial was judged to be at overall low risk of bias, and missing outcome data were the most prominent concern. The immediate cognitive transfer findings were sensitive to trials at high risk of bias and to highly influential studies.

Second, the studies differed substantially in participant diagnosis and MCI subtype, training content and dose, mode of delivery, comparator intensity, outcome measurement, and follow-up duration. However, the number of trials was insufficient to reliably identify the independent contribution of these factors. Although transfer classification was based on a prespecified coding manual and the relationship between the training targets and outcome measures, the framework requires reviewer judgement and has not been independently validated specifically for CCT trials in MCI. Complex multidomain interventions and multidimensional neuropsychological tests were particularly challenging to classify, and classification error could not be completely excluded. In addition, the original pre-consensus independent coding sheets were not archived in a form that allowed calculation of Cohen’s κ; consequently, empirical inter-rater agreement could not be quantified retrospectively, which limits formal validation of the transfer taxonomy. When exact covariance estimates were unavailable, within-trial aggregation also relied on a working assumption of an outcome correlation of ρ = 0.50, which may have influenced the variance and weighting of the composite effect estimates.

Finally, actual training dose, adherence, reasons for withdrawal, technical difficulties, and adverse events were reported using inconsistent definitions and denominators, limiting assessment of the acceptability, safety, and real-world scalability of CCT. Technical support requirements and equipment-related problems in home-based and remotely delivered training were particularly underreported. In addition, this review included only formally published reports in English, which may have resulted in the omission of studies published in other languages, unpublished trials, or grey literature and may have increased the risk of bias due to missing evidence. The PROSPERO record contained inconsistencies in some fields and differed from the final review in certain operational details; the relationship between the registered information and the final review methodology is documented in the Supplementary material. A complete citation-level full-text exclusion log with prospectively archived records of individual screening decisions was not available from the initial screening stage. Although the available full-text exclusion records and reasons for exclusion are reported in the Supplementary material, independent retrospective verification of every exclusion decision is therefore not possible.

## 5 Conclusion

The available evidence base is small and methodologically limited. Immediate cognitive transfer estimates were imprecise, heterogeneous, and sensitive to influential trials at high risk of bias. For sustained cognitive near and far transfer, pooled estimates at least 12 weeks after training cessation were close to the null, but only four to five trials contributed and no included trial was at overall low risk of bias. These findings indicate a lack of convincing evidence for a durable average cognitive transfer benefit rather than demonstrating that such effects are absent; modest beneficial or harmful effects cannot be excluded given the limited evidence base. Evidence for functional far transfer remains insufficient. Future research should include adequately powered multicentre randomised trials with longer follow-up, prespecified and independently verifiable transfer classifications, standardised real-world functional outcomes, and comprehensive reporting of actual training dose, adherence, safety, and clinically meaningful progression outcomes.

## Data Availability

The original contributions presented in the study are included in the article/Supplementary material, further inquiries can be directed to the corresponding author.

## References

[ref1] Albert M. S. DeKosky S. T. Dickson D. Dubois B. Feldman H. H. Fox N. C. . (2011). The diagnosis of mild cognitive impairment due to Alzheimer's disease: recommendations from the National Institute on Aging-Alzheimer's Association workgroups on diagnostic guidelines for Alzheimer's disease. Alzheimers Dement. 7, 270–279. doi: 10.1016/j.jalz.2011.03.008, 21514249 PMC3312027

[ref2] Antonenko D. Thams F. Grittner U. Uhrich J. Glöckner F. Li S. C. . (2022). Randomized trial of cognitive training and brain stimulation in non-demented older adults. Alzheimers Dement. (N Y). 8:e12262. doi: 10.1002/trc2.12262, 35229023 PMC8864498

[ref3] Baker L. D. Pa J. A. Katula J. A. Aslanyan V. Salmon D. P. Jacobs D. M. . (2025). Effects of exercise on cognition and Alzheimer's biomarkers in a randomized controlled trial of adults with mild cognitive impairment: the EXERT study. Alzheimers Dement. 21:e14586. doi: 10.1002/alz.14586, 40271888 PMC12019696

[ref4] Balshem H. Helfand M. Schünemann H. J. Oxman A. D. Kunz R. Brozek J. . (2011). GRADE guidelines: 3. Rating the quality of evidence. J. Clin. Epidemiol. 64, 401–406. doi: 10.1016/j.jclinepi.2010.07.015 21208779

[ref5] Barnett S. M. Ceci S. J. (2002). When and where do we apply what we learn? A taxonomy for far transfer. Psychol. Bull. 128, 612–637. doi: 10.1037/0033-2909.128.4.612, 12081085

[ref6] Ben Izhak S. Diedrich L. Antal A. Lavidor M. (2024). Beyond social engagement: cognitive training leads to greater cognitive improvement in older adults. Neuropsychol. Dev. Cogn. B Aging Neuropsychol. Cogn. 31, 556–574. doi: 10.1080/13825585.2023.2216927, 37221447

[ref7] Booth S. J. Brown L. J. E. Taylor J. R. Pobric G. (2023). Experimental investigation of training schedule on home-based working memory training in healthy older adults. Front. Psychol. 14:1165275. doi: 10.3389/fpsyg.2023.1165275, 37187566 PMC10175577

[ref8] Borella E. Cantarella A. Carretti B. De Lucia A. De Beni R. (2019). Improving everyday functioning in the old-old with working memory training. Am. J. Geriatr. Psychiatry 27, 975–983. doi: 10.1016/j.jagp.2019.01.210, 30878190

[ref9] Borenstein M. Hedges L. V. Higgins J. P. T. Rothstein H. R. (2009). Introduction to Meta-Analysis. Chichester: John Wiley & Sons.

[ref10] Broadhouse K. M. Singh M. F. Suo C. Gates N. Wen W. Brodaty H. . (2020). Hippocampal plasticity underpins long-term cognitive gains from resistance exercise in MCI. Neuroimage Clin. 25:102182. doi: 10.1016/j.nicl.2020.102182, 31978826 PMC6974789

[ref11] Cai X. Xu L. Zhang H. Sun T. Yu J. Jia X. . (2024). The effects of exergames for cognitive function in older adults with mild cognitive impairment: a systematic review and meta-analysis. Front. Neurol. 15:1424390. doi: 10.3389/fneur.2024.1424390, 39081342 PMC11286570

[ref12] Chan A. T. C. Ip R. T. F. Tran J. Y. S. Chan J. Y. C. Tsoi K. K. F. (2024). Computerized cognitive training for memory functions in mild cognitive impairment or dementia: a systematic review and meta-analysis. NPJ Digit. Med. 7:1. doi: 10.1038/s41746-023-00987-5, 38172429 PMC10764827

[ref13] Chen J. W. Du W. Q. Zhu K. (2024). Network meta-analysis of the effects of different cognitive trainings on the cognitive function of patients with mild cognitive impairment. J. Psychiatr. Res. 174, 26–45. doi: 10.1016/j.jpsychires.2024.03.051, 38608550

[ref14] Cong L. Ren Y. Wang Y. Hou T. Dong Y. Han X. . (2023). Mild cognitive impairment among rural-dwelling older adults in China: a community-based study. Alzheimers Dement. 19, 56–66. doi: 10.1002/alz.12629, 35262288 PMC10078715

[ref15] Dai X. Liu S. Li Y. Long S. Li X. Chen C. . (2024). White matter plasticity underpins cognitive gains after multidomain adaptive computerized cognitive training. J. Gerontol. A Biol. Sci. Med. Sci. 79:glae046. doi: 10.1093/gerona/glae046, 38387014

[ref16] Dowell-Esquivel C. Czaja S. J. Harvey P. D. Kallestrup P. Depp C. A. Saber J. N. (2024). Computerized cognitive and skills training in older people with mild cognitive impairment: using ecological momentary assessment to index treatment-related changes in real-world performance of technology-dependent functional tasks. Am. J. Geriatr. Psychiatry 32, 446–459. doi: 10.1016/j.jagp.2023.10.014, 37953132 PMC10950539

[ref17] Duff K. Ying J. Suhrie K. R. Dalley B. C. A. Atkinson T. J. Porter S. M. . (2022). Computerized cognitive training in amnestic mild cognitive impairment: a randomized clinical trial. J. Geriatr. Psychiatry Neurol. 35, 400–409. doi: 10.1177/08919887211006472, 33783254 PMC12860473

[ref18] Egger M. Davey Smith G. Schneider M. Minder C. (1997). Bias in meta-analysis detected by a simple, graphical test. BMJ 315, 629–634. doi: 10.1136/bmj.315.7109.629, 9310563 PMC2127453

[ref19] Fiatarone Singh M. A. Gates N. Saigal N. Wilson G. C. Meiklejohn J. Brodaty H. . (2014). The study of mental and resistance training (SMART) study—resistance training and/or cognitive training in mild cognitive impairment: a randomized, double-blind, double-sham controlled trial. J. Am. Med. Dir. Assoc. 15, 873–880. doi: 10.1016/j.jamda.2014.09.010, 25444575

[ref20] Formica C. Bonanno M. Sorbera C. Quartarone A. Giambò F. M. Marra A. . (2024). Smartphone-based cognitive Telerehabilitation: a usability and feasibility study focusing on mild cognitive impairment. Sensors (Basel) 24:525. doi: 10.3390/s24020525, 38257618 PMC10820398

[ref21] Gao Y. Liu N. (2025). Effects of digital technology-based serious games interventions for older adults with mild cognitive impairment: a meta-analysis of randomised controlled trials. Age Ageing 54:afaf080. doi: 10.1093/ageing/afaf080, 40192627

[ref22] Gates N. J. Vernooij R. W. Di Nisio M. Karim S. March E. Martínez G. . (2019). Computerised cognitive training for preventing dementia in people with mild cognitive impairment. Cochrane Database Syst. Rev. 3:CD012279. doi: 10.1002/14651858.CD012279.pub2, 30864747 PMC6415132

[ref23] GBD 2019 Dementia Forecasting Collaborators (2019). Estimation of the global prevalence of dementia in 2019 and forecasted prevalence in 2050: an analysis for the global burden of disease study 2019. Lancet Public Health 7, e105–e125. doi: 10.1016/S2468-2667(21)00249-8, 34998485 PMC8810394

[ref24] Givon Schaham N. Buckman Z. Rand D. (2024). TECH preserves global cognition of older adults with MCI compared with a control group: a randomized controlled trial. Aging Clin. Exp. Res. 36:1. doi: 10.1007/s40520-023-02659-6, 38252189 PMC10803538

[ref25] Guye S. von Bastian C. C. (2017). Working memory training in older adults: Bayesian evidence supporting the absence of transfer. Psychol. Aging 32, 732–746. doi: 10.1037/pag0000206, 29239658

[ref26] Harvey P. D. Strassnig A. Strassnig M. T. Rivera-Molina A. Mueller A. Depp C. A. . (2025). Awareness of baseline functioning and sensitivity to improvement in older people with and without mild cognitive impairment receiving a computerized functional skills training program. Int. Psychogeriatr. 37:100025. doi: 10.1016/j.inpsyc.2024.100025, 40500103

[ref27] He Z. Tian S. Singh A. Chakraborty S. Zhang S. Lustria M. L. A. . (2022). A machine-learning based approach for predicting older adults' adherence to technology-based cognitive training. Inf. Process. Manag. 59:103034. doi: 10.1016/j.ipm.2022.103034, 35909793 PMC9337718

[ref28] Hedges L. V. Olkin I. (1985). Statistical Methods for Meta-Analysis. Orlando: Academic Press.

[ref29] Hill N. T. Mowszowski L. Naismith S. L. Chadwick V. L. Valenzuela M. Lampit A. (2017). Computerized cognitive training in older adults with mild cognitive impairment or dementia: a systematic review and Meta-analysis. Am. J. Psychiatry 174, 329–340. doi: 10.1176/appi.ajp.2016.16030360, 27838936

[ref30] Hwang H. F. Tseng K. C. Chen S. J. Yu W. Y. Chen C. Y. Lin M. R. (2023). Effects of home-based computerized cognitive training and tai chi exercise on cognitive functions in older adults with mild cognitive impairment. Aging Ment. Health 27, 2170–2178. doi: 10.1080/13607863.2023.2225430, 37365961

[ref31] IntHout J. Ioannidis J. P. Rovers M. M. Goeman J. J. (2016). Plea for routinely presenting prediction intervals in meta-analysis. BMJ Open 6:e010247. doi: 10.1136/bmjopen-2015-010247, 27406637 PMC4947751

[ref32] Jeong H. S. Lee Y. W. Rhee T. G. Shim S. R. (2025). Efficacy of digital therapeutic applications for cognitive training among older adults with mild cognitive impairment or dementia: a systematic review and network meta-analysis of randomized controlled trials. Psychiatry Res. 348:116426. doi: 10.1016/j.psychres.2025.116426, 40073511

[ref33] Kawata N. Y. S. Nouchi R. Oba K. Matsuzaki Y. Kawashima R. (2022). Auditory cognitive training improves brain plasticity in healthy older adults: evidence from a randomized controlled trial. Front. Aging Neurosci. 14:826672. doi: 10.3389/fnagi.2022.826672, 35431898 PMC9010026

[ref34] Kim B. S. Jun S. Kim H. Disease Neuroimaging Initiative Alzheimer’s (2023). Cognitive trajectories and associated biomarkers in patients with mild cognitive impairment. J. Alzheimers Dis. 92, 803–814. doi: 10.3233/JAD-220326, 36806501

[ref35] Kim H. Jung J. Lee S. (2022). Therapeutic application of virtual reality in the rehabilitation of mild cognitive impairment: a systematic review and Meta-analysis. Vision (Basel) 6:68. doi: 10.3390/vision6040068, 36412649 PMC9680273

[ref36] Knapp G. Hartung J. (2003). Improved tests for a random effects meta-regression with a single covariate. Stat. Med. 22, 2693–2710. doi: 10.1002/sim.1482, 12939780

[ref37] Kua Z. Ong R. H. S. Chen N. Y. C. Yoon P. S. Chew S. T. H. Dong Y. . (2025). Virtual reality-based cognitive training for MCI in the elderly: a feasibility randomised pilot study. Ann. Acad. Med. Singap. 54, 445–447. doi: 10.47102/annals-acadmedsg.2024162, 40799097

[ref38] Kwan R. Y. C. Liu J. Sin O. S. K. Fong K. N. K. Qin J. Wong J. C. Y. . (2024). Effects of virtual reality motor-cognitive training for older people with cognitive frailty: multicentered randomized controlled trial. J. Med. Internet Res. 26:e57809. doi: 10.2196/57809, 39259959 PMC11425022

[ref39] Li R. Geng J. Yang R. Ge Y. Hesketh T. (2022). Effectiveness of computerized cognitive training in delaying cognitive function decline in people with mild cognitive impairment: systematic review and Meta-analysis. J. Med. Internet Res. 24:e38624. doi: 10.2196/38624, 36301590 PMC9650579

[ref40] Liu Y. Zheng M. Liu J. Cao C. Ding Y. (2023). Effects of cognitive training in flanker task on mobility of older adults: a randomized Controlled Trail. Gerontology 69, 593–602. doi: 10.1159/000529088, 36646052

[ref41] Manenti R. Gobbi E. Baglio F. Macis A. Ferrari C. Pagnoni I. . (2020). Effectiveness of an innovative cognitive treatment and telerehabilitation on subjects with mild cognitive impairment: a multicenter, randomized, active-controlled study. Front. Aging Neurosci. 12:585988. doi: 10.3389/fnagi.2020.585988, 33304267 PMC7701275

[ref42] Montero-Odasso M. Zou G. Speechley M. Almeida Q. J. Liu-Ambrose T. Middleton L. E. . (2023). Effects of exercise alone or combined with cognitive training and vitamin D supplementation to improve cognition in adults with mild cognitive impairment: a randomized clinical trial. JAMA Netw. Open 6:e2324465. doi: 10.1001/jamanetworkopen.2023.24465, 37471089 PMC10359965

[ref43] Motter J. N. Rushia S. N. Qian M. Ndouli C. Nwosu A. Petrella J. R. . (2024). Expectancy does not predict 18-month treatment outcomes with cognitive training in mild cognitive impairment. J. Prev Alzheimers Dis. 11, 71–78. doi: 10.14283/jpad.2023.62, 38230719 PMC10794849

[ref44] Nicastri C. M. McFeeley B. M. Simon S. S. Ledreux A. Håkansson K. Granholm A. C. . (2022). BDNF mediates improvement in cognitive performance after computerized cognitive training in healthy older adults. Alzheimers Dement. (N Y) 8:e12337. doi: 10.1002/trc2.12337, 36089933 PMC9428279

[ref45] Page M. J. McKenzie J. E. Bossuyt P. M. Boutron I. Hoffmann T. C. Mulrow C. D. . (2020). The PRISMA 2020 statement: an updated guideline for reporting systematic reviews. BMJ 372:n71. doi: 10.1136/bmj.n71, 33782057 PMC8005924

[ref46] Papaioannou T. Voinescu A. Petrini K. Stanton Fraser D. (2022). Efficacy and moderators of virtual reality for cognitive training in people with dementia and mild cognitive impairment: a systematic review and meta-analysis. J. Alzheimer's Dis 88, 1341–1370. doi: 10.3233/JAD-210672 35811514

[ref47] Petersen R. C. (2016). Mild cognitive impairment. Continuum (Minneap Minn) 22, 404–418. doi: 10.1212/CON.0000000000000313, 27042901 PMC5390929

[ref48] Pieruccini-Faria F. Son S. Zou G. Almeida Q. J. Middleton L. E. Bray N. W. . (2025). Synergistic effects of exercise, cognitive training and vitamin D on gait performance and falls in mild cognitive impairment-secondary outcomes from the SYNERGIC trial. Age Ageing 54:afaf242. doi: 10.1093/ageing/afaf242, 40966614 PMC12445844

[ref49] Qin Y. Han H. Li Y. Cui J. Jia H. Ge X. . (2023). Estimating bidirectional transitions and identifying predictors of mild cognitive impairment. Neurology 100, e297–e307. doi: 10.1212/WNL.0000000000201386, 36220593 PMC9869761

[ref50] Rajan K. B. Weuve J. Barnes L. L. McAninch E. A. Wilson R. S. Evans D. A. (2021). Population estimate of people with clinical Alzheimer's disease and mild cognitive impairment in the United States (2020-2060). Alzheimers Dement. 17, 1966–1975. doi: 10.1002/alz.12362, 34043283 PMC9013315

[ref51] Ren Y. Wang Q. Liu H. Wang G. Lu A. (2024). Effects of immersive and non-immersive virtual reality-based rehabilitation training on cognition, motor function, and daily functioning in patients with mild cognitive impairment or dementia: a systematic review and meta-analysis. Clin. Rehabil. 38, 305–321. doi: 10.1177/02692155231213476, 38289618

[ref52] Rethlefsen M. L. Kirtley S. Waffenschmidt S. Ayala A. P. Moher D. Page M. J. . (2021). PRISMA-S: an extension to the PRISMA statement for reporting literature searches in systematic reviews. Syst. Rev. 10:39. doi: 10.1186/s13643-020-01542-z, 33499930 PMC7839230

[ref53] Röver C. Knapp G. Friede T. (2015). Hartung-Knapp-Sidik-Jonkman approach and its modification for random-effects meta-analysis with few studies. BMC Med. Res. Methodol. 15:99. doi: 10.1186/s12874-015-0091-1, 26573817 PMC4647507

[ref54] Sheehy L. Sveistrup H. Knoefel F. Taillon-Hobson A. Martin T. Egan M. . (2022). The use of home-based nonimmersive virtual reality to encourage physical and cognitive exercise in people with mild cognitive impairment: a feasibility study. J. Aging Phys. Act. 30, 297–307. doi: 10.1123/japa.2021-0043, 34453024

[ref55] Sterne J. A. C. Savović J. Page M. J. Elbers R. G. Blencowe N. S. Boutron I. . (2019). RoB 2: a revised tool for assessing risk of bias in randomised trials. BMJ 366:l4898. doi: 10.1136/bmj.l4898, 31462531

[ref56] Sterne J. A. Sutton A. J. Ioannidis J. P. Terrin N. Jones D. R. Lau J. . (2011). Recommendations for examining and interpreting funnel plot asymmetry in meta-analyses of randomised controlled trials. BMJ 343, d4002–d4002. doi: 10.1136/bmj.d4002, 21784880

[ref57] Thomas K. R. Bangen K. J. Weigand A. J. Ortiz G. Walker K. S. Salmon D. P. . (2022). Cognitive heterogeneity and risk of progression in data-driven subtle cognitive decline phenotypes. J. Alzheimer's Dis 90, 323–331. doi: 10.3233/JAD-220684, 36120785 PMC9661321

[ref58] Tortora C. Di Crosta A. La Malva P. Prete G. Ceccato I. Mammarella N. . (2024). Virtual reality and cognitive rehabilitation for older adults with mild cognitive impairment: a systematic review. Ageing Res. Rev. 93:102146. doi: 10.1016/j.arr.2023.102146, 38036103

[ref59] Viechtbauer W. (2010). Conducting meta-analyses in R with the metafor package. J. Stat. Softw. 36, 1–48. doi: 10.18637/jss.v036.i03

[ref60] Winblad B. Palmer K. Kivipelto M. Jelic V. Fratiglioni L. Wahlund L. O. . (2004). Mild cognitive impairment--beyond controversies, towards a consensus: report of the international working group on mild cognitive impairment. J. Intern. Med. 256, 240–246. doi: 10.1111/j.1365-2796.2004.01380.x, 15324367

[ref61] Wu J. He Y. Liang S. Liu Z. Huang J. Liu W. . (2023). Effects of computerized cognitive training on structure-function coupling and topology of multiple brain networks in people with mild cognitive impairment: a randomized controlled trial. Alzheimer's Res. Ther. 15:158. doi: 10.1186/s13195-023-01292-9, 37742005 PMC10517473

[ref62] Yang Q. Zhang L. Chang F. Yang H. Chen B. Liu Z. (2025). Virtual reality interventions for older adults with mild cognitive impairment: systematic review and Meta-analysis of randomized controlled trials. J. Med. Internet Res. 27:e59195. doi: 10.2196/59195, 39793970 PMC11759915

[ref63] Ye Y. Lei M. Chen L. Song R. Zhao F. Zhang L. (2024). Efficacy of technology-based cognitive and exercise interventions for mild cognitive impairment: a systematic review, network meta-analysis, and meta-regression of randomized controlled trials. Ageing Res. Rev. 100:102438. doi: 10.1016/j.arr.2024.102438, 39069094

[ref64] Zhang H. Huntley J. Bhome R. Holmes B. Cahill J. Gould R. L. . (2019). Effect of computerised cognitive training on cognitive outcomes in mild cognitive impairment: a systematic review and meta-analysis. BMJ Open 9:e027062. doi: 10.1136/bmjopen-2018-027062, 31427316 PMC6701629

[ref65] Zhong D. Chen L. Feng Y. Song R. Huang L. Liu J. . (2021). Effects of virtual reality cognitive training in individuals with mild cognitive impairment: a systematic review and meta-analysis. Int. J. Geriatr. Psychiatry 36, 1829–1847. doi: 10.1002/gps.5603, 34318524

[ref66] Zou C. Amos-Richards D. Jagannathan R. Kulshreshtha A. (2024). Effect of home-based lifestyle interventions on cognition in older adults with mild cognitive impairment: a systematic review. BMC Geriatr. 24:200. doi: 10.1186/s12877-024-04798-5, 38413870 PMC10900825

[ref67] Zuschnegg J. Schoberer D. Häussl A. Herzog S. A. Russegger S. Ploder K. . (2023). Effectiveness of computer-based interventions for community-dwelling people with cognitive decline: a systematic review with meta-analyses. BMC Geriatr. 23:229. doi: 10.1186/s12877-023-03941-y, 37041494 PMC10091663

