# Supplementary material for: Cognitive transfer and durability of computerised cognitive training in older adults with mild cognitive impairment: a systematic review and meta-analysis of randomised controlled trials
Source: Front Aging Neurosci. 2026 Oct 15;18:1952783. doi: 10.3389/fnagi.2026.1952783 (PMC13716727; doi:10.3389/fnagi.2026.1952783)
Supplement: Supplementary file 1 [file Supplementary_file_1.pdf]

Supplementary Materials

PROSPERO CRD420261463612

This supplementary file provides additional methodological details, supporting analyses, and reproducibility information for the systematic review and meta-analysis.

The supplementary material includes complete search strategies, trial linkage information, outcome classification, effect-size calculations, study-level analytical data, sensitivity analyses, risk-of-bias assessments, certainty-of-evidence evaluations, study-selection audit information, and PROSPERO registration and deviation information.

Supplementary Table S1. Complete search strategies and records retrieved

Database	Final search date	Records retrieved
PubMed	1 July 2026	359
Web of Science Core Collection	1 July 2026	376
Embase	1 July 2026	574
Cochrane Library	1 July 2026	93
Scopus	1 July 2026	1419
Wiley Online Library	1 July 2026	893
CINAHL	1 July 2026	1336

The seven database yields sum to 5,050 records. Backward and forward citation searching identified no additional unique records.

PubMed

Final search date: 1 July 2026; records retrieved: 359.

```
("Mild Cognitive Impairment"[Mesh] OR "mild cognitive impairment"[Title/Abstract] OR "mild cognitive impairments"[Title/Abstract] OR "amnestic mild cognitive impairment"[Title/Abstract] OR "amnestic MCI"[Title/Abstract] OR aMCI[Title/Abstract] OR "nonamnestic mild cognitive impairment"[Title/Abstract] OR "non-amnestic mild cognitive impairment"[Title/Abstract] OR naMCI[Title/Abstract] OR "mild neurocognitive disorder"[Title/Abstract] OR "mild neurocognitive disorders"[Title/Abstract] OR "mild cognitive disorder"[Title/Abstract] OR "mild cognitive disorders"[Title/Abstract] OR "mild cognitive dysfunction"[Title/Abstract] OR MCI[Title/Abstract]) AND (("Cognitive Training"[Mesh] OR "cognitive training"[Title/Abstract] OR "computerized cognitive training"[Title/Abstract] OR "computerised cognitive training"[Title/Abstract] OR "computer based cognitive training"[Title/Abstract] OR "computer-based cognitive training"[Title/Abstract] OR "computer assisted cognitive training"[Title/Abstract] OR "computer-assisted cognitive training"[Title/Abstract] OR "digital cognitive training"[Title/Abstract] OR "online cognitive training"[Title/Abstract] OR "internet based cognitive training"[Title/Abstract] OR "internet-based cognitive training"[Title/Abstract] OR "web based cognitive training"[Title/Abstract] OR "web-based cognitive training"[Title/Abstract] OR "mobile cognitive training"[Title/Abstract] OR "app based cognitive training"[Title/Abstract] OR "app-based cognitive training"[Title/Abstract] OR "game based cognitive training"[Title/Abstract] OR "game-based cognitive training"[Title/Abstract] OR "brain training"[Title/Abstract] OR "memory training"[Title/Abstract] OR "working memory training"[Title/Abstract] OR "attention training"[Title/Abstract] OR "executive function training"[Title/Abstract] OR "executive training"[Title/Abstract] OR "processing speed training"[Title/Abstract] OR "cognitive rehabilitation"[Title/Abstract] OR "computerized cognitive rehabilitation"[Title/Abstract] OR "computerised cognitive rehabilitation"[Title/Abstract] OR "digital cognitive rehabilitation"[Title/Abstract] OR "cognitive remediation"[Title/Abstract] OR "computerized cognitive remediation"[Title/Abstract] OR "computerised cognitive remediation"[Title/Abstract] OR "cognitive stimulation"[Title/Abstract] OR "computerized cognitive stimulation"[Title/Abstract] OR "computerised cognitive stimulation"[Title/Abstract] OR "cognitive exercise"[Title/Abstract] OR "cognitive exercises"[Title/Abstract] OR "neurocognitive training"[Title/Abstract] OR "neuropsychological training"[Title/Abstract]) AND ("Therapy, Computer-Assisted"[Mesh] OR "Computer-Assisted Instruction"[Mesh] OR "Video Games"[Mesh] OR "Virtual Reality"[Mesh] OR "Mobile Applications"[Mesh] OR computer*[Title/Abstract] OR computerized[Title/Abstract] OR computerised[Title/Abstract] OR digital[Title/Abstract] OR electronic[Title/Abstract] OR internet[Title/Abstract] OR online[Title/Abstract] OR "web based"[Title/Abstract] OR "web-based"[Title/Abstract] OR tablet[Title/Abstract] OR tablets[Title/Abstract] OR smartphone[Title/Abstract] OR smartphones[Title/Abstract] OR "mobile application"[Title/Abstract] OR "mobile applications"[Title/Abstract] OR "mobile app"[Title/Abstract] OR "mobile apps"[Title/Abstract] OR software[Title/Abstract] OR "video game"[Title/Abstract] OR "video games"[Title/Abstract] OR videogame[Title/Abstract] OR videogames[Title/Abstract] OR "serious game"[Title/Abstract] OR "serious games"[Title/Abstract] OR "virtual reality"[Title/Abstract] OR telerehabilitation[Title/Abstract] OR "tele-rehabilitation"[Title/Abstract] OR "home based technology"[Title/Abstract] OR "home-based technology"[Title/Abstract])) AND (randomized controlled trial[Publication Type] OR controlled clinical trial[Publication Type] OR randomized[Title/Abstract] OR randomised[Title/Abstract] OR placebo[Title/Abstract] OR randomly[Title/Abstract] OR trial[Title/Abstract] OR groups[Title/Abstract]) NOT (animals[MeSH Terms] NOT humans[MeSH Terms])
```

Web of Science Core Collection

Final search date: 1 July 2026; records retrieved: 376.

```
TS=(("mild cognitive impairment" OR "mild cognitive impairments" OR "amnestic mild cognitive impairment" OR "amnestic MCI" OR aMCI OR "nonamnestic mild cognitive impairment" OR "non-amnestic mild cognitive impairment" OR naMCI OR "mild neurocognitive disorder*" OR "mild cognitive disorder*" OR "mild cognitive dysfunction" OR MCI) AND ("computerized cognitive training" OR "computerised cognitive training" OR "computer-based cognitive training" OR "computer based cognitive training" OR "computer-assisted cognitive training" OR "computer assisted cognitive training" OR "digital cognitive training" OR "online cognitive training" OR "internet-based cognitive training" OR "internet based cognitive training" OR "web-based cognitive training" OR "web based cognitive training" OR "mobile cognitive training" OR "app-based cognitive training" OR "app based cognitive training" OR "game-based cognitive training" OR "game based cognitive training" OR "computerized cognitive rehabilitation" OR "computerised cognitive rehabilitation" OR "computer-based cognitive rehabilitation" OR "computer assisted cognitive rehabilitation" OR "digital cognitive rehabilitation" OR "computerized cognitive remediation" OR "computerised cognitive remediation" OR "computerized memory training" OR "computerised memory training" OR "computerized working memory
```

training" OR "computerised working memory training" OR "computerized attention training" OR "computerised attention training" OR "computerized executive function training" OR "computerised executive function training" OR "computerized processing speed training" OR "computerised processing speed training" OR "brain training" OR (((cognit* OR memory OR "working memory" OR attention OR executive OR "executive function*" OR "processing speed" OR neurocognit* OR neuropsycholog*) NEAR/3 (train* OR rehabilitat* OR remediat* OR exercis*)) AND (computer* OR digital OR electronic OR online OR internet OR "web based" OR web-based OR tablet* OR smartphone* OR mobile OR app* OR software OR videogame* OR "video game*" OR "serious game*" OR "virtual reality" OR telerehabilitation OR tele-rehabilitation)) OR BrainHQ OR Cogmed OR Lumosity OR COGPACK OR RehaCom OR NeuroNation OR CogniFit OR "Brain Fitness Program" OR "Posit Science") AND (random* OR trial OR placebo OR "controlled stud*" OR "control group*" OR "parallel group*" OR "random allocation" OR "randomly assigned" OR "waitlist control*" OR "waiting list control*" OR "sham control*"))

Embase

Final search date: 1 July 2026; records retrieved: 574.

('mild cognitive impairment'/exp OR 'mild cognitive impairment':ti,ab,kw OR 'mild cognitive impairments':ti,ab,kw OR 'amnestic mild cognitive impairment':ti,ab,kw OR 'amnestic mild cognitive impairments':ti,ab,kw OR 'amnestic mci':ti,ab,kw OR amci:ti,ab,kw OR 'nonamnestic mild cognitive impairment':ti,ab,kw OR 'non-amnestic mild cognitive impairment':ti,ab,kw OR 'non amnestic mild cognitive impairment':ti,ab,kw OR namci:ti,ab,kw OR 'mild neurocognitive disorder':ti,ab,kw OR 'mild neurocognitive disorders':ti,ab,kw OR 'mild cognitive disorder':ti,ab,kw OR 'mild cognitive disorders':ti,ab,kw OR 'mild cognitive dysfunction':ti,ab,kw OR mci:ti,ab,kw) AND ((('cognitive rehabilitation'/exp OR 'cognitive therapy'/exp) AND ('computer'/exp OR 'computer assisted therapy'/exp OR 'mobile application'/exp OR 'internet'/exp OR 'virtual reality'/exp)) OR 'computerized cognitive training':ti,ab,kw OR 'computerised cognitive training':ti,ab,kw OR 'computer based cognitive training':ti,ab,kw OR 'computer-based cognitive training':ti,ab,kw OR 'computer assisted cognitive training':ti,ab,kw OR 'computer-assisted cognitive training':ti,ab,kw OR 'computerized cognitive rehabilitation':ti,ab,kw OR 'computerised cognitive rehabilitation':ti,ab,kw OR 'computer assisted cognitive rehabilitation':ti,ab,kw OR 'computer-assisted cognitive rehabilitation':ti,ab,kw OR 'computerized cognitive intervention':ti,ab,kw OR 'computerised cognitive intervention':ti,ab,kw OR 'digital cognitive training':ti,ab,kw OR 'digital cognitive intervention':ti,ab,kw OR 'online cognitive training':ti,ab,kw OR 'internet based cognitive training':ti,ab,kw OR 'internet-based cognitive training':ti,ab,kw OR 'web based cognitive training':ti,ab,kw OR 'web-based cognitive training':ti,ab,kw OR 'tablet based cognitive training':ti,ab,kw OR 'tablet-based cognitive training':ti,ab,kw OR 'mobile cognitive training':ti,ab,kw OR 'app based cognitive training':ti,ab,kw OR 'app-based cognitive training':ti,ab,kw OR 'game based cognitive training':ti,ab,kw OR 'game-based cognitive training':ti,ab,kw OR 'video game cognitive training':ti,ab,kw OR 'videogame cognitive training':ti,ab,kw OR 'brain training':ti,ab,kw OR 'cognitive training software':ti,ab,kw OR 'cognitive training program':ti,ab,kw OR 'cognitive training programme':ti,ab,kw OR ((computer* OR digital OR online OR internet OR web OR tablet* OR smartphone* OR mobile OR app OR apps OR software OR videogame* OR 'video game*') NEAR/3 ('cognitive train*' OR 'cognitive exercis*' OR 'cognitive rehabilitat*' OR 'cognitive remediat*' OR 'memory train*' OR 'attention train*' OR 'executive function train*' OR 'working memory train*')):ti,ab,kw OR (('virtual reality' OR 'virtual reality based' OR 'virtual-reality-based') NEAR/5 (cognit* OR memory OR attention OR executive) NEAR/5 (train* OR rehabilitat* OR intervention* OR therap*)):ti,ab,kw OR cogmed:ti,ab,kw OR brainhq:ti,ab,kw OR 'brain hq':ti,ab,kw OR lumosity:ti,ab,kw) AND ('randomized controlled trial'/exp OR 'randomization'/exp OR 'controlled clinical trial'/exp OR 'single blind procedure'/exp OR 'double blind procedure'/exp OR 'triple blind procedure'/exp OR 'crossover procedure'/exp OR random*:ti,ab,kw OR placebo*:ti,ab,kw OR allocat*:ti,ab,kw OR ((singl* OR doubl* OR tripl* OR trebl*) NEAR/2 (blind* OR mask*)):ti,ab,kw OR crossover*:ti,ab,kw OR 'cross over':ti,ab,kw OR ((control* OR clinical) NEAR/2 trial*):ti,ab,kw OR rct:ti,ab,kw) NOT ('animal'/exp NOT 'human'/exp)

Cochrane Library

Final search date: 1 July 2026; records retrieved: 93.

#1 MeSH descriptor: [Mild Cognitive Impairment] explode all trees
#2 ("mild cognitive impairment" OR "mild cognitive impairments" OR "amnestic mild cognitive impairment" OR "amnestic mild cognitive impairments" OR "amnestic MCI" OR aMCI):ti,ab,kw
#3 ("nonamnestic mild cognitive impairment" OR "non-amnestic mild cognitive impairment" OR "non amnestic mild cognitive impairment" OR "nonamnestic MCI" OR "non-amnestic MCI" OR naMCI):ti,ab,kw
#4 ("mild neurocognitive disorder" OR "mild neurocognitive disorders" OR "mild cognitive disorder" OR "mild cognitive disorders" OR "mild cognitive dysfunction"):ti,ab,kw
#5 ("cognitive impairment no dementia" OR "cognitive impairment without dementia" OR CIND OR MCI):ti,ab,kw
#6 #1 OR #2 OR #3 OR #4 OR #5
#7 MeSH descriptor: [Cognitive Training] explode all trees
#8 MeSH descriptor: [Cognitive Remediation] explode all trees
#9 MeSH descriptor: [Therapy, Computer-Assisted] explode all trees
#10 MeSH descriptor: [Computer-Assisted Instruction] explode all trees
#11 MeSH descriptor: [Computers, Handheld] explode all trees
#12 MeSH descriptor: [Mobile Applications] explode all trees
#13 MeSH descriptor: [Virtual Reality] explode all trees
#14 MeSH descriptor: [Video Games] explode all trees
#15 #7 OR #8
#16 #9 OR #10 OR #11 OR #12 OR #13 OR #14
#17 #15 AND #16
#18 ("computerized cognitive training" OR "computerised cognitive training" OR "computer-based cognitive training" OR "computer based cognitive training" OR "computer-assisted cognitive training" OR "computer assisted cognitive training" OR "computerized cognitive rehabilitation" OR "computerised cognitive rehabilitation" OR "computer-assisted cognitive rehabilitation" OR "computer assisted cognitive rehabilitation" OR "computerized cognitive remediation" OR "computerised cognitive remediation" OR "computer-assisted cognitive remediation" OR "computer assisted cognitive remediation" OR "computerized cognitive intervention" OR "computerised cognitive intervention"):ti,ab,kw
#19 ("digital cognitive training" OR "digital cognitive intervention" OR "digital cognitive rehabilitation" OR "online cognitive training" OR "online cognitive intervention" OR "internet-based cognitive training" OR "internet based cognitive training" OR "web-based cognitive training" OR "web based cognitive training" OR "tablet-based cognitive training" OR "tablet based cognitive

training" OR "tablet-based cognitive intervention" OR "mobile cognitive training" OR "mobile-based cognitive training" OR "smartphone-based cognitive training" OR "app-based cognitive training" OR "app based cognitive training" OR "software-based cognitive training"):ti,ab,kw
#20 ("game-based cognitive training" OR "game based cognitive training" OR "video game-based cognitive training" OR "video game cognitive training" OR "videogame-based cognitive training" OR "videogame cognitive training" OR "virtual reality cognitive training" OR "virtual reality-based cognitive training" OR "virtual reality cognitive rehabilitation" OR "virtual reality-based cognitive rehabilitation" OR "brain training" OR "computerized brain training" OR "computerised brain training" OR "cognitive training software" OR "cognitive training program" OR "cognitive training programme"):ti,ab,kw
#21 ((computer* OR digital OR online OR internet OR web* OR tablet* OR smartphone* OR mobile OR app* OR software OR videogame* OR "video game*" OR "virtual reality") NEAR/5 ((cognit* NEAR/2 (train* OR exercis* OR rehabilitat* OR remediat* OR intervention*)) OR (memory NEAR/2 train*) OR (attention NEAR/2 train*) OR ("executive function" NEAR/2 train*) OR ("working memory" NEAR/2 train*))):ti,ab,kw
#22 (Cogmed OR BrainHQ OR "Brain HQ" OR Lumosity OR CogniFit OR RehaCom OR NeuroNation OR HappyNeuron OR "Posit Science" OR "Brain Fitness Program"):ti,ab,kw
#23 #17 OR #18 OR #19 OR #20 OR #21 OR #22
#24 #6 AND #23

Scopus

Final search date: 1 July 2026; records retrieved: 1419.

TITLE-ABS-KEY("mild cognitive impairment" OR "amnestic mild cognitive impairment" OR "amnestic MCI" OR aMCI OR "nonamnestic mild cognitive impairment" OR "non-amnestic mild cognitive impairment" OR naMCI OR "mild neurocognitive disorder" OR "mild cognitive disorder" OR "mild cognitive dysfunction" OR MCI) AND TITLE-ABS-KEY("computerized cognitive training" OR "computerised cognitive training" OR "computer based cognitive training" OR "computer assisted cognitive training" OR "digital cognitive training" OR "online cognitive training" OR "internet based cognitive training" OR "web based cognitive training" OR "mobile cognitive training" OR "app based cognitive training" OR "game based cognitive training" OR "computerized cognitive rehabilitation" OR "computerised cognitive rehabilitation" OR "digital cognitive rehabilitation" OR "computerized cognitive remediation" OR "computerised cognitive remediation" OR "computerized cognitive stimulation" OR "computerised cognitive stimulation" OR "computerized memory training" OR "computerised memory training" OR "computerized working memory training" OR "computerised working memory training" OR "computerized attention training" OR "computerised attention training" OR "computerized executive function training" OR "computerised executive function training" OR "computerized processing speed training" OR "computerised processing speed training" OR "brain training" OR BrainHQ OR Cogmed OR Lumosity OR COGPACK OR RehaCom OR NeuroNation OR CogniFit OR "Brain Fitness Program" OR "Posit Science" OR ((cognit* OR memory OR "working memory" OR attention OR executive OR "executive function" OR "processing speed" OR neurocognit* OR neuropsycholog*) AND (train* OR rehabilitat* OR remediat* OR exercis*) AND (computer* OR digital OR electronic OR online OR internet OR "web based" OR tablet* OR touchscreen* OR "touch screen" OR smartphone* OR "mobile application" OR "mobile app" OR software OR videogame* OR "video game" OR "serious game" OR "virtual reality" OR telerehabilitation))) AND TITLE-ABS-KEY(random* OR trial OR RCT OR placebo OR "random allocation" OR "randomly assigned" OR "random assignment" OR "randomized controlled trial" OR "randomised controlled trial" OR "controlled clinical trial" OR "controlled trial" OR "controlled study" OR "parallel group" OR "control group" OR "waitlist control" OR "waiting list control" OR "sham control" OR crossover OR "cross over")

Wiley Online Library

Final search date: 1 July 2026; records retrieved: 893.

("mild cognitive impairment" OR "mild cognitive impairments" OR "amnestic mild cognitive impairment" OR "amnestic mild cognitive impairments" OR "amnestic MCI" OR aMCI OR "nonamnestic mild cognitive impairment" OR "non-amnestic mild cognitive impairment" OR "non amnestic mild cognitive impairment" OR "nonamnestic MCI" OR "non-amnestic MCI" OR naMCI OR "mild neurocognitive disorder" OR "mild neurocognitive disorders" OR "mild cognitive disorder" OR "mild cognitive disorders" OR "mild cognitive dysfunction" OR "cognitive impairment no dementia" OR "cognitive impairment without dementia" OR CIND OR MCI) AND ("computerized cognitive training" OR "computerised cognitive training" OR "computerized cognitive trainings" OR "computerised cognitive trainings" OR "computer-based cognitive training" OR "computer based cognitive training" OR "computer-assisted cognitive training" OR "computer assisted cognitive training" OR "computerized cognitive rehabilitation" OR "computerised cognitive rehabilitation" OR "computer-assisted cognitive rehabilitation" OR "computer assisted cognitive rehabilitation" OR "computerized cognitive remediation" OR "computerised cognitive remediation" OR "computer-assisted cognitive remediation" OR "computer assisted cognitive remediation" OR "computerized cognitive intervention" OR "computerised cognitive intervention" OR "digital cognitive training" OR "digital cognitive intervention" OR "digital cognitive rehabilitation" OR "online cognitive training" OR "online cognitive intervention" OR "internet-based cognitive training" OR "internet based cognitive training" OR "web-based cognitive training" OR "web based cognitive training" OR "tablet-based cognitive training" OR "tablet based cognitive training" OR "tablet-based cognitive intervention" OR "mobile cognitive training" OR "mobile-based cognitive training" OR "smartphone-based cognitive training" OR "app-based cognitive training" OR "application-based cognitive training" OR "software-based cognitive training" OR "game-based cognitive training" OR "game based cognitive training" OR "video game-based cognitive training" OR "video game cognitive training" OR "videogame-based cognitive training" OR "videogame cognitive training" OR "virtual reality cognitive training" OR "virtual reality-based cognitive training" OR "virtual reality cognitive rehabilitation" OR "virtual reality-based cognitive rehabilitation" OR "brain training" OR "computerized brain training" OR "computerised brain training" OR "cognitive training software" OR "cognitive training program" OR "cognitive training programme" OR "computerized memory training" OR "computerised memory training" OR "computer-based memory training" OR "computerized attention training" OR "computerised attention training" OR "computerized executive function training" OR "computerised executive function training" OR "computerized working memory training" OR "computerised working memory training" OR Cogmed OR BrainHQ OR "Brain HQ" OR Lumosity OR CogniFit OR RehaCom OR NeuroNation OR HappyNeuron OR "Posit Science" OR "Brain Fitness Program") AND ("randomized controlled trial" OR "randomised controlled trial" OR "controlled clinical trial" OR "randomized clinical trial" OR "randomised clinical trial" OR "clinical trial" OR "controlled trial" OR randomized OR randomised OR randomly OR randomization OR randomisation OR allocation OR allocated OR placebo OR "single blind" OR "single-blind" OR "double blind" OR "double-blind" OR "triple blind" OR "triple-blind" OR masked OR masking OR crossover OR "cross-over" OR "parallel group" OR "parallel-group" OR RCT)

CINAHL

Final search date: 1 July 2026; records retrieved: 1336.

((MH "Mild Cognitive Impairment+") OR TI ("mild cognitive impairment" OR "mild cognitive impairments" OR "amnestic mild cognitive impairment" OR "amnestic mild cognitive impairments" OR "amnestic MCI" OR aMCI OR "nonamnestic mild cognitive impairment" OR "non-amnestic mild cognitive impairment" OR "non amnestic mild cognitive impairment" OR naMCI OR "mild neurocognitive disorder" OR "mild neurocognitive disorders" OR "mild cognitive disorder" OR "mild cognitive disorders" OR "mild cognitive dysfunction" OR "cognitive impairment no dementia" OR CIND OR MCI) OR AB ("mild cognitive impairment" OR "mild cognitive impairments" OR "amnestic mild cognitive impairment" OR "amnestic mild cognitive impairments" OR "amnestic MCI" OR aMCI OR "nonamnestic mild cognitive impairment" OR "non-amnestic mild cognitive impairment" OR "non amnestic mild cognitive impairment" OR naMCI OR "mild neurocognitive disorder" OR "mild neurocognitive disorders" OR "mild cognitive disorder" OR "mild cognitive disorders" OR "mild cognitive dysfunction" OR "cognitive impairment no dementia" OR CIND OR MCI)) AND (((MH "Cognitive Therapy+") OR (MH "Cognitive Rehabilitation") OR (MH "Computer Assisted Instruction+") OR (MH "Computers+") OR (MH "Virtual Reality+") OR (MH "Mobile Applications+") OR (MH "Internet+")) AND (TI (cognit* OR memory OR attention OR executive OR neuropsychological) OR AB (cognit* OR memory OR attention OR executive OR neuropsychological))) OR TI ("computerized cognitive training" OR "computerised cognitive training" OR "computer-based cognitive training" OR "computer based cognitive training" OR "computer-assisted cognitive training" OR "computer assisted cognitive training" OR "computerized cognitive rehabilitation" OR "computerised cognitive rehabilitation" OR "computer-assisted cognitive rehabilitation" OR "computer assisted cognitive rehabilitation" OR "computerized cognitive intervention" OR "computerised cognitive intervention" OR "digital cognitive training" OR "digital cognitive intervention" OR "online cognitive training" OR "internet-based cognitive training" OR "internet based cognitive training" OR "web-based cognitive training" OR "web based cognitive training" OR "tablet-based cognitive training" OR "tablet based cognitive training" OR "mobile cognitive training" OR "app-based cognitive training" OR "app based cognitive training" OR "game-based cognitive training" OR "game based cognitive training" OR "video game-based cognitive training" OR "videogame cognitive training" OR "virtual reality cognitive training" OR "virtual reality-based cognitive training" OR "brain training" OR "cognitive training software" OR "cognitive training program" OR "cognitive training programme" OR Cogmed OR BrainHQ OR "Brain HQ" OR Lumosity OR CogniFit OR RehaCom OR NeuroNation OR HappyNeuron OR "Posit Science" OR "Brain Fitness Program") OR AB ("computerized cognitive training" OR "computerised cognitive training" OR "computer-based cognitive training" OR "computer based cognitive training" OR "computer-assisted cognitive training" OR "computer assisted cognitive training" OR "computerized cognitive rehabilitation" OR "computerised cognitive rehabilitation" OR "computer-assisted cognitive rehabilitation" OR "computer assisted cognitive rehabilitation" OR "computerized cognitive intervention" OR "computerised cognitive intervention" OR "digital cognitive training" OR "digital cognitive intervention" OR "online cognitive training" OR "internet-based cognitive training" OR "internet based cognitive training" OR "web-based cognitive training" OR "web based cognitive training" OR "tablet-based cognitive training" OR "tablet based cognitive training" OR "mobile cognitive training" OR "app-based cognitive training" OR "app based cognitive training" OR "game-based cognitive training" OR "game based cognitive training" OR "video game-based cognitive training" OR "videogame cognitive training" OR "virtual reality cognitive training" OR "virtual reality-based cognitive training" OR "brain training" OR "cognitive training software" OR "cognitive training program" OR "cognitive training programme" OR Cogmed OR BrainHQ OR "Brain HQ" OR Lumosity OR CogniFit OR RehaCom OR NeuroNation OR HappyNeuron OR "Posit Science" OR "Brain Fitness Program") OR TI (((computer* OR digital OR online OR internet OR web OR tablet* OR smartphone* OR mobile OR app OR apps OR software OR videogame* OR "video game" OR "video games" OR "virtual reality") N3 ("cognitive training" OR "cognitive exercises" OR "cognitive rehabilitation" OR "cognitive remediation" OR "memory training" OR "attention training" OR "executive function training" OR "working memory training"))) OR AB (((computer* OR digital OR online OR internet OR web OR tablet* OR smartphone* OR mobile OR app OR apps OR software OR videogame* OR "video game" OR "video games" OR "virtual reality") N3 ("cognitive training" OR "cognitive exercises" OR "cognitive rehabilitation" OR "cognitive remediation" OR "memory training" OR "attention training" OR "executive function training" OR "working memory training")))) AND (((MH "Randomized Controlled Trials") OR (MH "Double-Blind Studies") OR (MH "Single-Blind Studies") OR (MH "Random Assignment") OR (MH "Pretest-Posttest Design") OR (MH "Cluster Sample") OR TI (randomised OR randomized OR trial) OR AB random* OR ((MH "Sample Size") AND AB (assigned OR allocated OR control)) OR (MH "Placebos") OR PT "Randomized Controlled Trial" OR AB (control W5 group) OR (MH "Crossover Design") OR (MH "Comparative Studies") OR AB (cluster W3 RCT)) NOT (((MH "Animals+") OR (MH "Animal Studies") OR TI "animal model*") NOT (MH "Human")))

Supplementary Table S2. Linkage of 11 reports to eight independent randomised trials

Trial_ID	Trial/family	Contributing reports	Country	Report role	Models contributed
SMART_ACTRN12608000489392	SMART	Fiatarone Singh et al. (2014); Broadhouse et al. (2020)	Australia	Primary trial report plus long-term secondary report	M1–M6
DUFF_NCT02301546	Duff trial	Duff et al. (2022)	United States	Primary and follow-up outcomes in one report	M1–M6
NCT02955277	TECH trial	Givon Schaham et al. (2024)	Israel	Primary trial report	M1–M4
NCT04679441	CCT+FUNSAT trial	Dowell-Esquivel et al. (2024); Harvey et al. (2025)	United States	Real-world performance and secondary awareness report	M2 and M5; 30-day outcomes classified as early maintenance
KUA_VRCT_2025	MindGym VR pilot	Kua et al. (2025)	Singapore	Pilot trial report	M1 and M2
NCT03486704	VRRS trial	Manenti et al. (2020)	Italy	Primary trial report	M1–M4
NCT02808676	SYNERGIC	Montero-Odasso et al. (2023); Pieruccini-Faria et al. (2025)	Canada	Cognitive primary report plus gait/falls secondary report	M2 and M4; gait/falls outside prespecified transfer models
ChiCTR2000034012	Wu trial	Wu et al. (2023)	China	Cognitive and neuroimaging report	M1 and M2

Reports from the same randomised sample were linked by Trial_ID and counted once in each model. Pieruccini-Faria et al. (2025) reported gait and falls outcomes from SYNERGIC that were outside the prespecified cognitive and functional transfer models.

Supplementary Table S3. Detailed characteristics of the included trials

Trial/reports	Country and setting	Participants and diagnosis	Randomised/analysed sample and age	CCT and comparator	Dose and delivery	Outcomes and follow-up
Duff, 2022	United States; academic centre; home-based	Clinically diagnosed single- or multidomain amnestic MCI; ≥65 years	Randomised 73/73; analysed 55/58 post-intervention and 43/51 at 1 year; mean age 74.9/74.9 years	Adaptive BrainHQ auditory processing, attention, and memory training vs non-adaptive visual computer games	45 min, 4–5 times/week; target 40 h over approximately 12–16 weeks	RBANS memory/attention and total score; ADCS-ADL-MCI; post-intervention and 1 year
SMART: Fiatarone Singh, 2014; Broadhouse, 2020	Australia; university research centre; supervised groups	Petersen-defined MCI; community-dwelling adults ≥55 years	CT 24; PRT 22; CT+PRT 27; double-sham 27; overall mean age 70.1 years	COGPACK multidomain CCT alone or with progressive resistance training vs sham cognitive training	2–3 times/week for 6 months; 60–100 min/session; supervised	ADAS-Cog, Bayer-ADL, cognitive-domain composites; 6 and 18 months
Givon Schaham, 2024	Israel; two community geriatric clinics; home/group-based	MCI; MoCA 19–25; confirmed memory complaint; independent ADL/IADL; >65 years	Randomised 30/31; analysed 25/25 post-intervention and 17/14 at 6 months; median age 74.5/74.0 years	Tablet-based TECH games and functional apps vs occupational-therapy advice or social board games	30–60 min, 3–5 times/week for 5 weeks; weekly group guidance	MoCA and WebNeuro cognitive domains; post-intervention and 6 months
Dowell-Esquivel, 2024; Harvey, 2025	United States; 14 community centres; remotely delivered at home	Jak–Bondi-defined MCI; MoCA ≥18; ≥60 years	Randomised 54/55; 47/45 completed training; mean age 71.7 years	BrainHQ processing-speed training followed by adaptive FUNSAT vs FUNSAT alone	Twice weekly for up to 12 weeks; CCT during the first 3 weeks	BAC-App, VRFCAT, FUNSAT, WHOQOL-BREF, MDPQ; post-intervention and 30 days after cessation
Kua, 2025	Singapore; hospital memory clinic	Petersen-defined MCI; MMSE ≥23; 65–85 years	Randomised 11/11; analysed 11/9 post-intervention	Immersive Oculus Go/MindGym multidomain VR training vs usual care plus cognitive-stimulation exercises	60 min once weekly for 10 weeks	MoCA, FAB, RBANS memory/visuospatial tests and inhibitory control; 10 weeks
Manenti, 2020	Italy; three rehabilitation centres; outpatient/home-based	Petersen-defined MCI; MMSE 24–30; CDR=0.5; >65 years	Three arms: 18/14/17; mean ages 75.3/76.3/78.1 years	Individualised multidomain VRRS followed by home VRRS or unstructured stimulation vs usual cognitive rehabilitation	Outpatient 60 min, 3 times/week for 4 weeks; home 60 min, 3 times/week for 12 weeks	Memory, executive, language, visuospatial and ADL/IADL outcomes; 1, 4, and 7 months
SYNERGIC: Montero-Odasso, 2023; Pieruccini-Faria, 2025	Canada; five university-affiliated centres	Subjective concerns plus objective cognitive impairment; function preserved; 60–85 years	Five groups: 34/35/37/35/34; mean ages 72.4–73.8 years	Adaptive CCT plus aerobic/resistance exercise vs sham CCT with exercise; vitamin D/placebo factorial design	90 min, 3 times/week for 20 weeks (30 min CCT/sham; 60 min exercise)	ADAS-Cog-13/Plus, cognitive domains and IADL; post-intervention, 6 and 12 months
Wu, 2023	China; three university hospitals; inpatient/outpatient	Petersen-defined MCI; MoCA ≤25 (≤24 with <12 years education); GDS 2–3; 50–85 years	Randomised 30/30; clinical assessment 29/28; final imaging analysis 25/25; mean age 67.7/65.5 years	Adaptive 11-module multidomain CCT vs one-to-one health education	60 min, 3 times/week for 8 weeks; 24 sessions	MoCA, DSST, Stroop, CAVLT, Rey complex figure recall; within 1 week post-intervention

ADL, activities of daily living; CCT, computerised cognitive training; CDR, Clinical Dementia Rating; FAB, Frontal Assessment Battery; IADL, instrumental activities of daily living; MCI, mild cognitive impairment; MoCA, Montreal Cognitive Assessment; RBANS, Repeatable Battery for the Assessment of Neuropsychological Status; VR, virtual reality.

Supplementary Table S4. Outcome-level effect inputs used in within-trial aggregation

Model	Trial/report	Outcome	Domain	Time window	Effect source	n	Hedges g	SE	95% CI
M1	Fiatarone Singh et al. (2014); Broadhouse et al. (2020)	Executive Function Domain	Executive function	Immediate	Author-reported bias-corrected COGNITIVE×TIME Hedges effect	100	-0.070	0.135	-0.330 to 0.200
M1	Fiatarone Singh et al. (2014); Broadhouse et al. (2020)	Memory Domain	Episodic memory	Immediate	Author-reported bias-corrected COGNITIVE×TIME Hedges effect	100	0.230	0.148	-0.060 to 0.520
M1	Fiatarone Singh et al. (2014); Broadhouse et al. (2020)	SDMT	Processing speed	Immediate	Author-reported bias-corrected COGNITIVE×TIME Hedges effect	100	-0.030	0.199	-0.420 to 0.360
M1	Duff et al. (2022)	Auditory Memory/Attention Index	Auditory memory/attention	Immediate	Baseline-constrained cLDA contrast, standardised by pooled baseline SD	113	-0.249	0.101	-0.448 to -0.050
M1	Kua et al. (2025)	RBANS List recall	Verbal memory	Immediate	Author-reported between-group change MD/95% CI, standardised by pooled baseline SD	20	0.485	0.337	-0.176 to 1.146
M1	Kua et al. (2025)	RBANS Figure recall	Visual memory	Immediate	Author-reported between-group change MD/95% CI, standardised by pooled baseline SD	20	0.482	0.282	-0.080 to 1.024
M1	Kua et al. (2025)	FAB Conflicting instructions	Executive function	Immediate	Author-reported between-group change MD/95% CI, standardised by pooled baseline SD	20	0.410	0.454	-0.547 to 1.231
M1	Kua et al. (2025)	FAB Go/no-go	Executive function	Immediate	Author-reported between-group change MD/95% CI, standardised by pooled baseline SD	20	0.914	0.412	0.061 to 1.675
M1	Manenti et al. (2020)	RAVLT immediate recall	Verbal memory	Immediate	Endpoint means and SDs, converted to direction-harmonised Hedges g	32	-0.035	0.356	-0.733 to 0.664
M1	Manenti et al. (2020)	RAVLT delayed recall	Verbal memory	Immediate	Endpoint means and SDs, converted to direction-harmonised Hedges g	32	-0.310	0.359	-1.013 to 0.393
M1	Manenti et al. (2020)	FCSRT immediate free recall	Verbal memory	Immediate	Endpoint means and SDs, converted to direction-harmonised Hedges g	32	-0.499	0.362	-1.208 to 0.211
M1	Manenti et al. (2020)	FCSRT immediate total recall	Verbal memory	Immediate	Endpoint means and SDs, converted to direction-harmonised Hedges g	32	-0.494	0.362	-1.204 to 0.215
M1	Manenti et al. (2020)	FCSRT delayed free recall	Verbal memory	Immediate	Endpoint means and SDs, converted to direction-harmonised Hedges g	32	-0.824	0.372	-1.552 to -0.095
M1	Manenti et al. (2020)	FCSRT delayed total recall	Verbal memory	Immediate	Endpoint means and SDs, converted to direction-harmonised Hedges g	32	-0.523	0.363	-1.234 to 0.188
M1	Manenti et al. (2020)	FCSRT index of sensitivity of cueing	Verbal memory	Immediate	Endpoint means and SDs, converted to direction-harmonised Hedges g	32	-0.593	0.364	-1.308 to 0.121

Model	Trial/report	Outcome	Domain	Time window	Effect source	n	Hedges g	SE	95% CI
M1	Manenti et al. (2020)	Trail Making Test A	Processing speed	Immediate	Endpoint means and SDs, converted to direction-harmonised Hedges g	32	0.300	0.358	-0.402 to 1.003
M1	Manenti et al. (2020)	Trail Making Test B	Executive function	Immediate	Endpoint means and SDs, converted to direction-harmonised Hedges g	32	0.155	0.357	-0.545 to 0.854
M1	Manenti et al. (2020)	Clock Drawing Test	Visuoconstruction	Immediate	Endpoint means and SDs, converted to direction-harmonised Hedges g	32	0.843	0.373	0.113 to 1.573
M1	Wu et al. (2023)	DSST	Processing speed	Immediate	Change-score means and SDs, converted to Hedges g	50	0.256	0.284	-0.301 to 0.812
M1	Wu et al. (2023)	Stroop C-A response time	Executive function	Immediate	Change-score means and SDs, converted to Hedges g	50	0.537	0.288	-0.027 to 1.102
M1	Wu et al. (2023)	CAVLT immediate recall	Verbal memory	Immediate	Change-score means and SDs, converted to Hedges g	50	0.752	0.293	0.178 to 1.327
M1	Wu et al. (2023)	CAVLT delayed recall	Verbal memory	Immediate	Change-score means and SDs, converted to Hedges g	50	0.662	0.291	0.092 to 1.232
M1	Wu et al. (2023)	Rey CFT recall	Visual memory	Immediate	Change-score means and SDs, converted to Hedges g	50	0.706	0.292	0.134 to 1.278
M1	Givon Schaham et al. (2024)	Working memory	Working memory	Immediate	Five-number summaries converted using Luo/Wan methods; endpoint SMD	61	0.281	0.257	-0.223 to 0.786
M2	Fiatarone Singh et al. (2014); Broadhouse et al. (2020)	ADAS-Cog	Global cognition	Immediate	Author-reported bias-corrected COGNITIVE×TIME Hedges effect	100	-0.070	0.199	-0.460 to 0.320
M2	Duff et al. (2022)	RBANS Total Scale	Global cognition	Immediate	Baseline-constrained cLDA contrast, standardised by pooled baseline SD	113	-0.203	0.120	-0.438 to 0.031
M2	Dowell-Esquivel et al. (2024); Harvey et al. (2025)	BAC-App composite	Global cognition	Immediate	Endpoint means and SDs	92	-0.347	0.210	-0.759 to 0.065
M2	Kua et al. (2025)	MoCA	Global cognition	Immediate	Author-reported between-group change MD/95% CI, standardised by pooled baseline SD	20	-0.203	0.421	-1.014 to 0.637
M2	Kua et al. (2025)	RBANS Figure copy	Visuoconstruction	Immediate	Author-reported between-group change MD/95% CI, standardised by pooled baseline SD	20	1.511	0.590	0.331 to 2.645
M2	Kua et al. (2025)	FAB total	Executive function	Immediate	Author-reported between-group change MD/95% CI, standardised by pooled baseline SD	20	0.294	0.387	-0.490 to 1.028
M2	Manenti et al. (2020)	Verbal fluency phonemic (FPL)	Language	Immediate	Endpoint means and SDs, converted to direction-harmonised Hedges g	32	-0.180	0.357	-0.880 to 0.520
M2	Manenti et al. (2020)	Verbal fluency semantic	Language	Immediate	Endpoint means and SDs,	32	0.320	0.359	-0.383 to 1.023

Model	Trial/report	Outcome	Domain	Time window	Effect source	n	Hedges g	SE	95% CI
		(FPC)			converted to direction-harmonised Hedges g				
M2	Manenti et al. (2020)	BADA object naming	Language	Immediate	Endpoint means and SDs, converted to direction-harmonised Hedges g	32	0.118	0.357	-0.582 to 0.817
M2	Manenti et al. (2020)	BADA action naming	Language	Immediate	Endpoint means and SDs, converted to direction-harmonised Hedges g	32	0.179	0.357	-0.521 to 0.879
M2	Wu et al. (2023)	MoCA	Global cognition	Immediate	Change-score means and SDs, converted to Hedges g	50	1.975	0.347	1.294 to 2.656
M2	Givon Schaham et al. (2024)	MoCA	Global cognition	Immediate	Five-number summaries converted using Luo/Wan methods; endpoint SMD	61	0.395	0.259	-0.112 to 0.902
M2	Givon Schaham et al. (2024)	Sustained attention	Attention	Immediate	Five-number summaries converted using Luo/Wan methods; endpoint SMD	61	0.130	0.256	-0.372 to 0.633
M2	Givon Schaham et al. (2024)	Controlled attention	Attention	Immediate	Five-number summaries converted using Luo/Wan methods; endpoint SMD	61	0.040	0.256	-0.462 to 0.542
M2	Givon Schaham et al. (2024)	Flexibility	Executive function	Immediate	Five-number summaries converted using Luo/Wan methods; endpoint SMD	61	0.216	0.257	-0.287 to 0.720
M2	Givon Schaham et al. (2024)	Inhibition	Executive function	Immediate	Five-number summaries converted using Luo/Wan methods; endpoint SMD	61	0.524	0.261	0.014 to 1.035
M2	Givon Schaham et al. (2024)	Memory recall	Episodic memory	Immediate	Five-number summaries converted using Luo/Wan methods; endpoint SMD	61	0.082	0.256	-0.420 to 0.584
M2	Givon Schaham et al. (2024)	Problem-solving	Executive function	Immediate	Five-number summaries converted using Luo/Wan methods; endpoint SMD	61	-0.268	0.257	-0.773 to 0.236
M2	Montero-Odasso et al. (2023); Pieruccini-Faria et al. (2025)	ADAS-Cog-13	Global cognition	Immediate	Author-adjusted MD and reported Cohen d	118	0.387	0.186	0.023 to 0.752
M3	Fiatarone Singh et al. (2014); Broadhouse et al. (2020)	Executive Function Domain	Executive function	Sustained (≥12 weeks)	Author-reported bias-corrected COGNITIVE×TIME Hedges effect	100	-0.090	0.135	-0.360 to 0.170
M3	Fiatarone Singh et al. (2014); Broadhouse et al. (2020)	Memory Domain	Episodic memory	Sustained (≥12 weeks)	Author-reported bias-corrected COGNITIVE×TIME Hedges effect	100	0.120	0.148	-0.170 to 0.410
M3	Fiatarone Singh et al. (2014); Broadhouse et al. (2020)	SDMT	Processing speed	Sustained (≥12 weeks)	Author-reported bias-corrected COGNITIVE×TIME Hedges effect	100	-0.140	0.199	-0.530 to 0.250
M3	Duff et al. (2022)	Auditory Memory/Attention Index	Auditory memory/attention	Sustained (≥12 weeks)	Baseline-constrained cLDA contrast, standardised by pooled baseline SD	94	-0.122	0.115	-0.347 to 0.102
M3	Manenti et al. (2020)	RAVLT immediate recall	Verbal memory	Sustained (≥12 weeks)	Endpoint means and SDs, converted to direction-harmonised Hedges g	32	-0.894	0.375	-1.629 to -0.160

Model	Trial/report	Outcome	Domain	Time window	Effect source	n	Hedges g	SE	95% CI
M3	Manenti et al. (2020)	RAVLT delayed recall	Verbal memory	Sustained (≥12 weeks)	Endpoint means and SDs, converted to direction-harmonised Hedges g	32	-0.629	0.365	-1.346 to 0.087
M3	Manenti et al. (2020)	FCSRT immediate free recall	Verbal memory	Sustained (≥12 weeks)	Endpoint means and SDs, converted to direction-harmonised Hedges g	32	-0.482	0.362	-1.191 to 0.227
M3	Manenti et al. (2020)	FCSRT immediate total recall	Verbal memory	Sustained (≥12 weeks)	Endpoint means and SDs, converted to direction-harmonised Hedges g	32	-0.551	0.363	-1.263 to 0.161
M3	Manenti et al. (2020)	FCSRT delayed free recall	Verbal memory	Sustained (≥12 weeks)	Endpoint means and SDs, converted to direction-harmonised Hedges g	32	-0.391	0.360	-1.097 to 0.314
M3	Manenti et al. (2020)	FCSRT delayed total recall	Verbal memory	Sustained (≥12 weeks)	Endpoint means and SDs, converted to direction-harmonised Hedges g	32	-0.573	0.364	-1.287 to 0.140
M3	Manenti et al. (2020)	FCSRT index of sensitivity of cueing	Verbal memory	Sustained (≥12 weeks)	Endpoint means and SDs, converted to direction-harmonised Hedges g	32	-0.593	0.364	-1.308 to 0.121
M3	Manenti et al. (2020)	Trail Making Test A	Processing speed	Sustained (≥12 weeks)	Endpoint means and SDs, converted to direction-harmonised Hedges g	32	0.352	0.359	-0.352 to 1.056
M3	Manenti et al. (2020)	Trail Making Test B	Executive function	Sustained (≥12 weeks)	Endpoint means and SDs, converted to direction-harmonised Hedges g	32	0.249	0.358	-0.453 to 0.950
M3	Manenti et al. (2020)	Clock Drawing Test	Visuoconstruction	Sustained (≥12 weeks)	Endpoint means and SDs, converted to direction-harmonised Hedges g	32	1.038	0.381	0.292 to 1.785
M3	Givon Schaham et al. (2024)	Working memory	Working memory	Sustained (≥12 weeks)	Five-number summaries converted using Luo/Wan methods; endpoint SMD	61	0.064	0.256	-0.438 to 0.566
M4	Fiatarone Singh et al. (2014); Broadhouse et al. (2020)	ADAS-Cog	Global cognition	Sustained (≥12 weeks)	Author-reported bias-corrected COGNITIVE×TIME Hedges effect	100	-0.180	0.199	-0.570 to 0.210
M4	Duff et al. (2022)	RBANS Total Scale	Global cognition	Sustained (≥12 weeks)	Baseline-constrained cLDA contrast, standardised by pooled baseline SD	94	-0.193	0.129	-0.446 to 0.060
M4	Manenti et al. (2020)	Verbal fluency phonemic (FPL)	Language	Sustained (≥12 weeks)	Endpoint means and SDs, converted to direction-harmonised Hedges g	32	0.035	0.356	-0.664 to 0.733
M4	Manenti et al. (2020)	Verbal fluency semantic (FPC)	Language	Sustained (≥12 weeks)	Endpoint means and SDs, converted to direction-harmonised Hedges g	32	0.223	0.358	-0.478 to 0.923
M4	Manenti et al. (2020)	BADA object naming	Language	Sustained (≥12 weeks)	Endpoint means and SDs, converted to direction-harmonised Hedges g	32	-0.134	0.357	-0.833 to 0.566
M4	Manenti et al. (2020)	BADA action naming	Language	Sustained (≥12 weeks)	Endpoint means and SDs, converted to direction-harmonised Hedges g	32	-0.035	0.356	-0.733 to 0.664
M4	Givon Schaham et al. (2024)	MoCA	Global cognition	Sustained (≥12 weeks)	Five-number summaries converted using Luo/Wan methods; endpoint SMD	61	0.533	0.261	0.022 to 1.044
M4	Givon Schaham et al.	Sustained attention	Attention	Sustained (≥12 weeks)	Five-number summaries	61	0.186	0.257	-0.317 to 0.689

Model	Trial/report	Outcome	Domain	Time window	Effect source	n	Hedges g	SE	95% CI
	(2024)				converted using Luo/Wan methods; endpoint SMD				
M4	Givon Schaham et al. (2024)	Controlled attention	Attention	Sustained (≥12 weeks)	Five-number summaries converted using Luo/Wan methods; endpoint SMD	61	0.429	0.259	-0.079 to 0.937
M4	Givon Schaham et al. (2024)	Flexibility	Executive function	Sustained (≥12 weeks)	Five-number summaries converted using Luo/Wan methods; endpoint SMD	61	0.292	0.258	-0.212 to 0.797
M4	Givon Schaham et al. (2024)	Inhibition	Executive function	Sustained (≥12 weeks)	Five-number summaries converted using Luo/Wan methods; endpoint SMD	61	0.485	0.260	-0.024 to 0.995
M4	Givon Schaham et al. (2024)	Memory recall	Episodic memory	Sustained (≥12 weeks)	Five-number summaries converted using Luo/Wan methods; endpoint SMD	61	0.068	0.256	-0.434 to 0.570
M4	Givon Schaham et al. (2024)	Problem-solving	Executive function	Sustained (≥12 weeks)	Five-number summaries converted using Luo/Wan methods; endpoint SMD	61	-0.150	0.256	-0.653 to 0.353
M4	Montero-Odasso et al. (2023); Pieruccini-Faria et al. (2025)	ADAS-Cog-13	Global cognition	Sustained (≥12 weeks)	Derived between-group model change, standardised by pooled baseline SD	110	0.151	0.103	-0.051 to 0.354
M5	Fiatarone Singh et al. (2014); Broadhouse et al. (2020)	BAYER-ADL	Daily function	Immediate	Author-reported bias-corrected COGNITIVE×TIME Hedges effect	100	-0.310	0.202	-0.700 to 0.090
M5	Duff et al. (2022)	ADCS-ADL-MCI	Daily function	Immediate	Baseline-constrained cLDA contrast, standardised by pooled baseline SD	113	-0.153	0.189	-0.525 to 0.217
M5	Dowell-Esquivel et al. (2024); Harvey et al. (2025)	VRFCAT total time	Functional capacity	Immediate	Endpoint means and SDs	92	-0.012	0.209	-0.420 to 0.397
M6	Fiatarone Singh et al. (2014); Broadhouse et al. (2020)	BAYER-ADL	Daily function	Sustained (≥12 weeks)	Author-reported bias-corrected COGNITIVE×TIME Hedges effect	100	-0.270	0.199	-0.660 to 0.120
M6	Duff et al. (2022)	ADCS-ADL-MCI	Daily function	Sustained (≥12 weeks)	Baseline-constrained cLDA contrast, standardised by pooled baseline SD	94	0.051	0.204	-0.349 to 0.450

Only outcomes retained in the prespecified primary within-trial aggregation are shown. Author-reported domain composites were used in preference to their component measures when this avoided duplicate weighting. Positive values favour CCT.

Supplementary Table S5. Trial-level composite effects used in models M1–M6

Model	Outcome model	Trial/report	n	Domains included	Hedges g (95% CI)	Variance	Working ρ
M1	Immediate cognitive near transfer	Fiatarone Singh et al. (2014); Broadhouse et al. (2020)	100	Episodic memory; Executive function; Processing speed	0.05 (-0.19 to 0.29)	0.0149	0.50
M1	Immediate cognitive near transfer	Duff et al. (2022)	113	Auditory memory/attention	-0.25 (-0.45 to -0.05)	0.0103	0.50
M1	Immediate cognitive near transfer	Kua et al. (2025)	20	Executive function (2 measures); Verbal memory; Visual memory	0.51 (0.01 to 1.02)	0.0672	0.50
M1	Immediate cognitive near transfer	Manenti et al. (2020)	32	Executive function; Processing speed; Verbal memory (7 measures); Visuoconstruction	-0.11 (-0.61 to 0.39)	0.0648	0.50
M1	Immediate cognitive near transfer	Wu et al. (2023)	50	Executive function; Processing speed; Verbal memory (2 measures); Visual memory	0.58 (0.15 to 1.00)	0.0476	0.50
M1	Immediate cognitive near transfer	Givon Schaham et al. (2024)	61	Working memory	0.28 (-0.22 to 0.79)	0.0663	0.50
M2	Immediate cognitive far transfer	Fiatarone Singh et al. (2014); Broadhouse et al. (2020)	100	Global cognition	-0.07 (-0.46 to 0.32)	0.0396	0.50
M2	Immediate cognitive far transfer	Duff et al. (2022)	113	Global cognition	-0.20 (-0.44 to 0.03)	0.0143	0.50
M2	Immediate cognitive far transfer	Dowell-Esquivel et al. (2024); Harvey et al. (2025)	92	Global cognition	-0.35 (-0.76 to 0.06)	0.0442	0.50
M2	Immediate cognitive far transfer	Kua et al. (2025)	20	Executive function; Global cognition; Visuoconstruction	0.11 (-0.58 to 0.79)	0.1214	0.50
M2	Immediate cognitive far transfer	Manenti et al. (2020)	32	Language (4 measures)	0.11 (-0.45 to 0.66)	0.0798	0.50
M2	Immediate cognitive far transfer	Wu et al. (2023)	50	Global cognition	1.97 (1.29 to 2.66)	0.1206	0.50
M2	Immediate cognitive far transfer	Givon Schaham et al. (2024)	61	Attention (2 measures); Episodic memory; Executive function (3 measures); Global cognition	0.15 (-0.21 to 0.51)	0.0332	0.50
M2	Immediate cognitive far transfer	Montero-Odasso et al. (2023); Pieruccini-Faria et al. (2025)	118	Global cognition	0.39 (0.02 to 0.75)	0.0345	0.50
M3	Sustained cognitive near transfer	Fiatarone Singh et al. (2014); Broadhouse et al. (2020)	100	Episodic memory; Executive function; Processing speed	-0.01 (-0.25 to 0.23)	0.0149	0.50
M3	Sustained cognitive near transfer	Duff et al. (2022)	94	Auditory memory/attention	-0.12 (-0.35 to 0.10)	0.0131	0.50
M3	Sustained cognitive near transfer	Manenti et al. (2020)	32	Executive function; Processing speed; Verbal memory (7 measures); Visuoconstruction	-0.16 (-0.66 to 0.34)	0.0656	0.50
M3	Sustained cognitive near transfer	Givon Schaham et al. (2024)	61	Working memory	0.06 (-0.44 to 0.57)	0.0656	0.50
M4	Sustained cognitive far transfer	Fiatarone Singh et al. (2014); Broadhouse et al. (2020)	100	Global cognition	-0.18 (-0.57 to 0.21)	0.0396	0.50
M4	Sustained cognitive far transfer	Duff et al. (2022)	94	Global cognition	-0.19 (-0.45 to 0.06)	0.0166	0.50

Model	Outcome model	Trial/report	n	Domains included	Hedges g (95% CI)	Variance	Working ρ
M4	Sustained cognitive far transfer	Manenti et al. (2020)	32	Language (4 measures)	0.02 (-0.53 to 0.57)	0.0795	0.50
M4	Sustained cognitive far transfer	Givon Schaham et al. (2024)	61	Attention (2 measures); Episodic memory; Executive function (3 measures); Global cognition	0.26 (-0.10 to 0.61)	0.0334	0.50
M4	Sustained cognitive far transfer	Montero-Odasso et al. (2023); Pieruccini-Faria et al. (2025)	110	Global cognition	0.15 (-0.05 to 0.35)	0.0106	0.50
M5	Immediate functional far transfer	Fiatarone Singh et al. (2014); Broadhouse et al. (2020)	100	Daily function	-0.31 (-0.70 to 0.08)	0.0406	0.50
M5	Immediate functional far transfer	Duff et al. (2022)	113	Daily function	-0.15 (-0.52 to 0.22)	0.0358	0.50
M5	Immediate functional far transfer	Dowell-Esquivel et al. (2024); Harvey et al. (2025)	92	Functional capacity	-0.01 (-0.42 to 0.40)	0.0435	0.50
M6	Sustained functional far transfer	Fiatarone Singh et al. (2014); Broadhouse et al. (2020)	100	Daily function	-0.27 (-0.66 to 0.12)	0.0396	0.50
M6	Sustained functional far transfer	Duff et al. (2022)	94	Daily function	0.05 (-0.35 to 0.45)	0.0416	0.50

Within each Trial_ID, eligible outcomes were first aggregated within domain and then across domains. Non-diagonal covariance terms were specified as $Cov(g_j,g_k)=\rho\sqrt{(v_j v_k)}$.

Supplementary Table S6. Sensitivity analyses

S6A. Alternative within-trial working correlations

Model	ρ	k	N	Hedges g (95% CI)	P value	I^2	τ^2
M1	0.30	6	376	0.15 (-0.22 to 0.52)	0.351	77.9%	0.092
M1	0.50	6	376	0.14 (-0.23 to 0.50)	0.373	72.9%	0.081
M1	0.70	6	376	0.12 (-0.23 to 0.48)	0.418	67.8%	0.069
M2	0.30	8	586	0.24 (-0.33 to 0.82)	0.352	83.8%	0.374
M2	0.50	8	586	0.23 (-0.35 to 0.81)	0.382	83.6%	0.382
M2	0.70	8	586	0.20 (-0.39 to 0.80)	0.447	83.7%	0.401
M3	0.30	4	287	-0.07 (-0.30 to 0.16)	0.398	0.0%	0.000
M3	0.50	4	287	-0.07 (-0.31 to 0.18)	0.452	0.0%	0.000
M3	0.70	4	287	-0.06 (-0.31 to 0.19)	0.500	0.0%	0.000
M4	0.30	5	397	0.03 (-0.24 to 0.30)	0.804	48.5%	0.023
M4	0.50	5	397	0.02 (-0.26 to 0.29)	0.881	42.9%	0.021
M4	0.70	5	397	0.01 (-0.27 to 0.28)	0.954	38.3%	0.019
M5	0.30	3	305	-0.16 (-0.66 to 0.33)	0.296	0.0%	0.000
M5	0.50	3	305	-0.16 (-0.66 to 0.33)	0.296	0.0%	0.000
M5	0.70	3	305	-0.16 (-0.66 to 0.33)	0.296	0.0%	0.000

Changing ρ did not affect M5 because each contributing trial supplied one eligible functional far-transfer effect. M6 remained unpooled because only two independent trials were available.

S6B. Exclusion of trials at overall high risk of bias

Model	k	N	Hedges g (95% CI)	P value	I^2	τ^2
M1	4	265	0.00 (-0.48 to 0.49)	0.981	67.3%	0.055
M2	5	383	0.04 (-0.32 to 0.39)	0.798	47.0%	0.038
M3	3	226	-0.08 (-0.42 to 0.26)	0.430	0.0%	0.000
M4	4	336	-0.04 (-0.38 to 0.31)	0.759	42.3%	0.020
M5	2	213	Not pooled	—	—	—

Overall high-risk trials were the Dowell-Esquivel/Harvey trial, Wu et al. (2023), and Givon Schaham et al. (2024). M5 was not re-pooled after exclusion because only two trials remained.

Supplementary Table S7. Leave-one-out influence analyses

Model	Omitted trial	Remaining k	Remaining N	Pooled Hedges g (95% CI)
M1	Fiatarone Singh et al. (2014)	5	276	0.17 (-0.32 to 0.67)
M1	Duff et al. (2022)	5	263	0.24 (-0.12 to 0.60)
M1	Kua et al. (2025)	5	356	0.08 (-0.33 to 0.49)
M1	Manenti et al. (2020)	5	344	0.19 (-0.27 to 0.64)
M1	Wu et al. (2023)	5	326	0.04 (-0.32 to 0.41)
M1	Givon Schaham et al. (2024)	5	315	0.12 (-0.33 to 0.58)
M2	Fiatarone Singh et al. (2014)	7	486	0.28 (-0.41 to 0.97)
M2	Duff et al. (2022)	7	473	0.30 (-0.37 to 0.98)
M2	Kua et al. (2025)	7	566	0.25 (-0.44 to 0.94)
M2	Manenti et al. (2020)	7	554	0.25 (-0.44 to 0.95)
M2	Wu et al. (2023)	7	536	0.00 (-0.26 to 0.25)
M2	Givon Schaham et al. (2024)	7	525	0.25 (-0.45 to 0.95)
M2	Dowell-Esquivel et al. (2024)	7	494	0.32 (-0.34 to 0.97)
M2	Montero-Odasso et al. (2023)	7	468	0.21 (-0.49 to 0.91)
M3	Fiatarone Singh et al. (2014)	3	187	-0.10 (-0.52 to 0.32)
M3	Duff et al. (2022)	3	193	-0.02 (-0.46 to 0.41)
M3	Manenti et al. (2020)	3	255	-0.06 (-0.40 to 0.29)
M3	Givon Schaham et al. (2024)	3	226	-0.08 (-0.42 to 0.26)
M4	Fiatarone Singh et al. (2014)	4	297	0.05 (-0.30 to 0.41)
M4	Duff et al. (2022)	4	303	0.11 (-0.14 to 0.36)
M4	Manenti et al. (2020)	4	365	0.01 (-0.34 to 0.37)
M4	Givon Schaham et al. (2024)	4	336	-0.04 (-0.38 to 0.31)
M4	Montero-Odasso et al. (2023)	4	287	-0.04 (-0.42 to 0.33)
M5	Fiatarone Singh et al. (2014)	2	205	-0.09 (-1.87 to 1.69)
M5	Duff et al. (2022)	2	192	-0.17 (-2.06 to 1.73)
M5	Dowell-Esquivel et al. (2024)	2	213	-0.23 (-1.98 to 1.53)
M6	Not performed: omitting one of two trials would leave a single study.	—	—	—

M5 reanalyses contained only two trials and are presented as influence diagnostics rather than stable formal sensitivity estimates. No leave-one-out analysis was performed for M6.

Supplementary Table S9. Trial-level RoB 2 judgements

Trial	D1	D2	D3	D4	D5	Overall	Principal reason
Fiatarone Singh et al. (2014)	Low	Low	Some concerns	Low	Low	Some concerns	Follow-up attrition and reliance on repeated-measures assumptions.
Duff et al. (2022)	Low	Low	Some concerns	Low	Low	Some concerns	Post-intervention and 1-year attrition; adjusted analysis reduced but did not eliminate concern.
Dowell-Esquivel/Harvey trial	Some concerns	High	High	Low	Some concerns	High	Discrepancies between randomised and analysed samples, missing outcome data, and limited information on adherence/deviations.
Kua et al. (2025)	Some concerns	Some concerns	Some concerns	Low	Some concerns	Some concerns	Pilot trial with limited reporting of randomisation, missing data, and outcome selection.
Manenti et al. (2020)	Some concerns	Some concerns	Low	Some concerns	Some concerns	Some concerns	Incomplete reporting of randomisation, outcome measurement, and selective reporting.
Wu et al. (2023)	Some concerns	Some concerns	High	Low	Some concerns	High	Complete-case analysis of 50/60 randomised participants.
Givon Schaham et al. (2024)	Low	Some concerns	High	Low	Some concerns	High	Only 31/61 participants completed 6-month follow-up; LOCF was used.
Montero-Odasso et al. (2023)	Low	Low	Some concerns	Low	Low	Some concerns	Some missing outcome data, but otherwise low risk across domains.

D1, bias arising from the randomisation process; D2, bias due to deviations from intended interventions; D3, bias due to missing outcome data; D4, bias in measurement of the outcome; D5, bias in selection of the reported result.
Overall: five trials had some concerns and three were at high risk; none was at low risk.

Supplementary Table S10. Detailed GRADE judgements

Model	Evidence base	Effect estimate	Risk of bias	Inconsistency	Indirectness	Imprecision	Bias due to missing evidence	Certainty	Rationale
M1	6 RCTs (N=376)	g=0.14 (95% CI −0.23 to 0.50); PI −0.74 to 1.02	Serious (−1)	Serious (−1)	Not serious	Serious (−1)	Not detected; no downgrade	Very low (⊕◯◯◯)	High-risk trials materially influenced the estimate; heterogeneity was substantial and both the confidence and prediction intervals included meaningful benefit and harm.
M2	8 RCTs (N=586)	g=0.23 (95% CI −0.35 to 0.81); PI −1.39 to 1.85	Serious (−1)	Serious (−1)	Not serious	Very serious (−2)	Not detected; no downgrade	Very low (⊕◯◯◯)	Wu et al. strongly influenced the point estimate and heterogeneity. The interval was compatible with marked benefit and harm.
M3	4 RCTs (N=287)	g=−0.07 (95% CI −0.31 to 0.18); PI −0.39 to 0.26	Not serious	Not serious	Not serious	Serious (−1)	Not detected; no downgrade	Moderate (⊕⊕⊕◯)	The estimate was stable after exclusion of high-risk trials and no statistically detectable heterogeneity was observed; certainty was downgraded for imprecision.
M4	5 RCTs (N=397)	g=0.02 (95% CI −0.26 to 0.29); PI −0.54 to 0.57	Not serious	Not serious	Not serious	Serious (−1)	Not detected; no downgrade	Moderate (⊕⊕⊕◯)	Excluding high-risk trials had little influence. The confidence interval remained compatible with small beneficial and harmful effects.
M5	3 RCTs (N=305)	g=−0.16 (95% CI −0.66 to 0.33); PI −1.62 to 1.30	Not serious	Not serious	Not serious	Very serious (−2)	Not detected; no downgrade	Low (⊕⊕◯◯)	Only three trials were available and the confidence and prediction intervals were very wide.
M6	2 RCTs (N=194)	Not pooled; individual g values −0.27 and 0.05	Not serious	Not serious*	Not serious	Very serious (−2)	Not detected; no downgrade	Low (⊕⊕◯◯)	Only two trials were available and the prespecified pooling threshold was not met. *Inconsistency could not be estimated reliably and was not automatically downgraded.

Randomised evidence started at high certainty and was downgraded only when justified. All models contained fewer than 10 trials; the inability to conduct Egger regression was not treated as automatic evidence of publication bias.

Figure S3A. Exploratory stratification by training delivery mode for immediate cognitive near transfer.

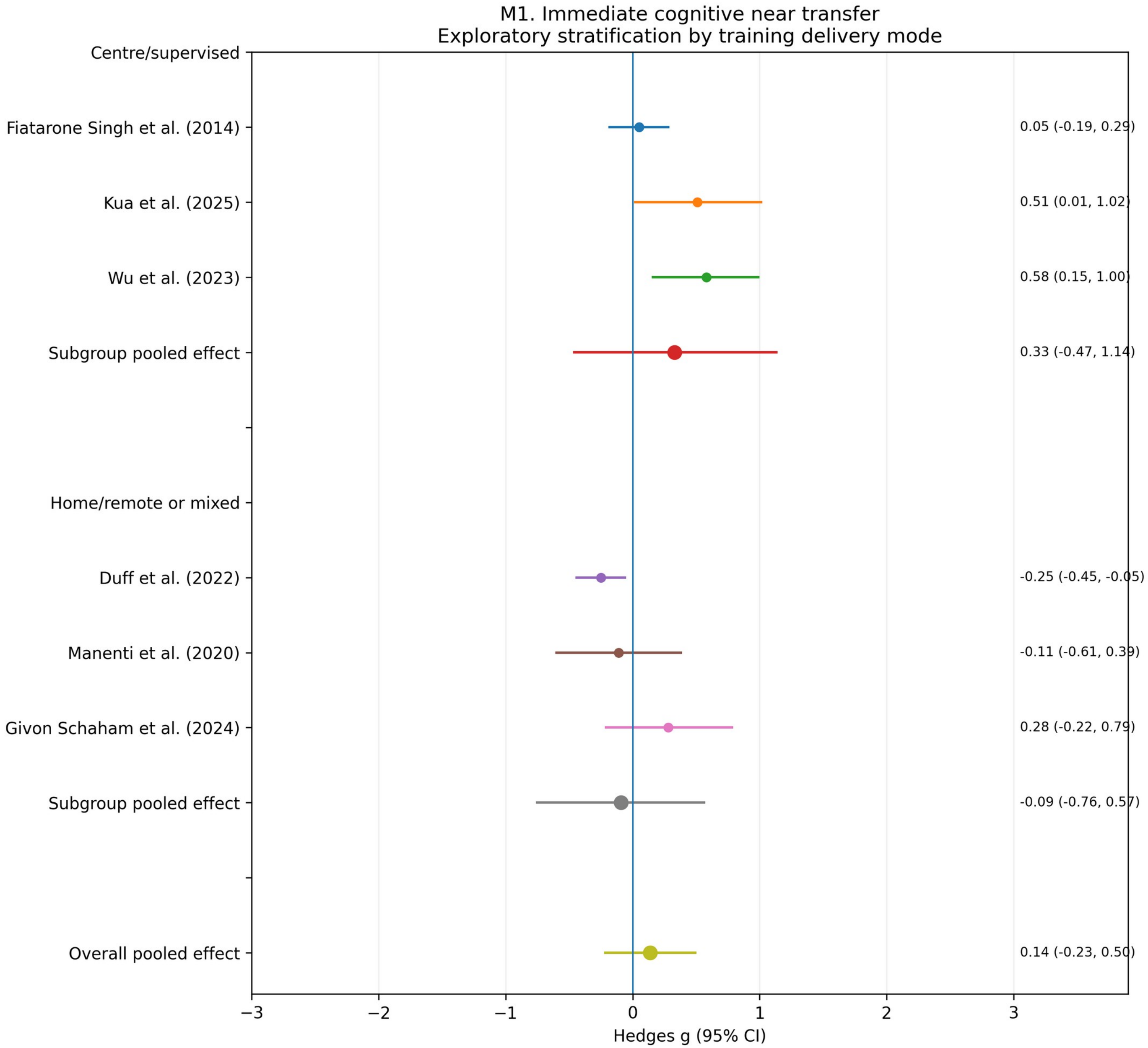

Figure S3B. Exploratory stratification by training delivery mode for immediate cognitive far transfer.

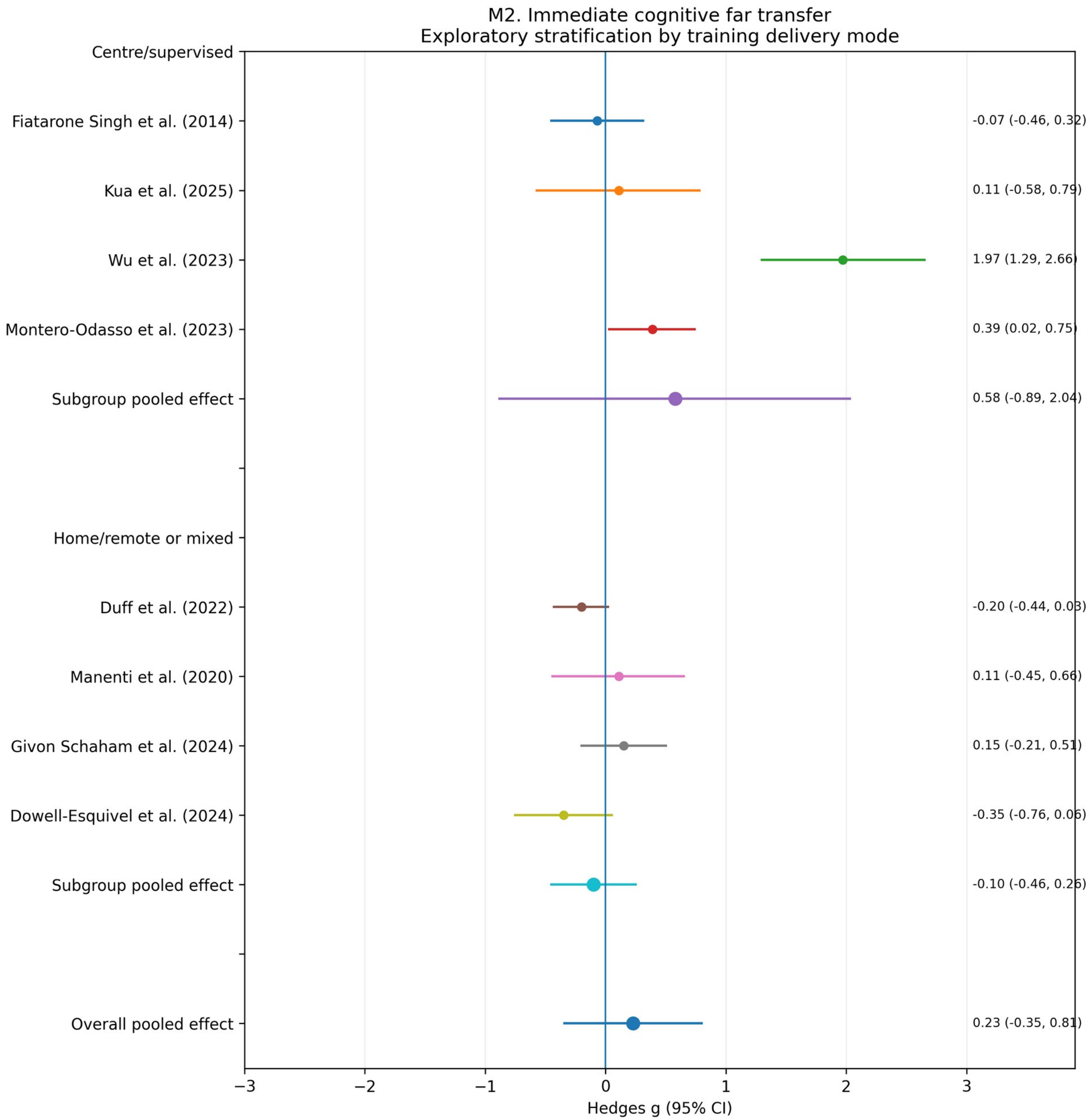

Supplementary Table S11. Training dose and comparator taxonomy

Trial/reports	Planned dose and delivery	Planned exposure	Comparator	Operational category	Analytic handling
SMART (Fiatarone Singh 2014; Broadhouse 2020)	2-3 sessions/week for 6 months; 60-100 min/session	Approximately 52-78 planned sessions	Sham cognitive training / common PRT factorial components	sham_CCT / factorial main effect	Dose and comparator were coded as potential effect modifiers; meta-analytic weights remained inverse-variance based.
Duff 2022	45 min, 4-5 sessions/week; target 40 h over approximately 12-16 weeks	Target approximately 40 h	Non-adaptive visual computer games	active_cognitive / sham-like	Active computer control reduces platform/contact differences.
Givon Schaham 2024	30-60 min, 3-5 sessions/week for 5 weeks	Approximately 15-25 sessions	Occupational-therapy advice or social board games	attention / active_cognitive	Comparator intensity varied within the control condition.
Dowell-Esquivel 2024; Harvey 2025	Twice weekly for up to 12 weeks; CCT delivered during first 3 weeks	6 CCT sessions; both arms continued FUNSAT	FUNSAT alone	add_on	Estimates the incremental effect of CCT added to the shared functional-skills intervention.
Kua 2025	60 min once weekly for 10 weeks	10 planned sessions	Usual care plus cognitive-stimulation exercises	active_cognitive	The source data support 10 planned weekly sessions, not 6.
Manenti 2020	Outpatient: 60 min, 3/week for 4 weeks; home phase: 60 min, 3/week for 12 weeks	12 clinic sessions plus up to 36 home sessions in the extended arm	Usual cognitive rehabilitation / unstructured stimulation	active_cognitive	Multiple phases and active comparison required timepoint-specific coding.
SYNERGIC (Montero-Odasso 2023; Pieruccini-Faria 2025)	90 min, 3/week for 20 weeks (30 min CCT/sham + 60 min exercise)	Approximately 60 CCT/sham sessions	Sham CCT with common exercise; factorial vitamin D/placebo	add_on / sham_CCT	CCT incremental effect identified against shared exercise.
Wu 2023	60 min, 3/week for 8 weeks	24 sessions	One-to-one health education	attention	Passive/educational comparator; influential in M2.

Interpretation: trial weights were determined by the precision of each trial-level effect estimate, not by training duration. Training dose and comparator type were prespecified as potential effect modifiers, but no primary model contained at least 10 independent trials; therefore, formal meta-regression or interaction testing was not performed. The previously reported delivery-mode stratification remains post hoc and hypothesis-generating.

Supplementary Table S12. Time-window and repeated-assessment rules applied in the final analysis

Supplementary Table S12A. Time-window rules applied in the final analysis

Time window	Operational definition	Primary selection rule	Analytic role
Immediate	End of final training session to <=4 weeks	Select the first complete assessment closest to training cessation; never select by statistical significance.	M1, M2, M5
Early maintenance	>4 weeks and <12 weeks after training cessation	Retain in All_effects/supportive evidence; do not substitute for the defined sustained window.	Supportive only
Sustained	>=12 weeks after training cessation	Select the longest eligible follow-up for each Trial_ID and transfer construct; record actual follow-up interval.	M3, M4, M6
Long-term sustained	>=24 weeks after training cessation	Pool separately only if at least 3 independent trials are available for the same transfer construct; otherwise summarise narratively.	Conditional/supportive

Supplementary Table S12B. Examples of repeated-assessment handling

Trial	Reported assessment	Mapped window	Final handling
Dowell-Esquivel/Harvey	30 days after cessation	Early maintenance	Excluded from M6 because it is <12 weeks; retained as supportive evidence.
Manenti	T1: after 4-week clinic phase	Intermediate during intervention	Not treated as final immediate endpoint because training continued in the home phase.
Manenti	T2: end of 12-week home phase (approximately 4 months from baseline)	Immediate	Selected for M1/M2 as the end-of-training assessment.
Manenti	T3: 3 months after home training stopped (approximately 7 months from baseline)	Sustained	Eligible for M3/M4.
SMART	6 months (end of intervention)	Immediate	Selected as the post-training assessment.
SMART	18 months (approximately 12 months after cessation)	Sustained	Selected as sustained follow-up.
Duff	Post-training after approximately 14-16 weeks of training	Immediate	Selected as post-training assessment.
Duff	1-year follow-up	Sustained	Selected as longest eligible follow-up.
Givon Schaham	5 weeks (end of intervention)	Immediate	Selected as post-training assessment.
Givon Schaham	6 months	Sustained	Selected as longest eligible follow-up.

Supplementary Table S13. Study-selection audit and eligibility transparency

Supplementary Table S13A. PRISMA numerical audit

PRISMA stage	n
Records identified from seven databases	5,050
Duplicate records removed	2,123
Records screened	2,927
Records excluded at title/abstract screening	2,805
Reports sought for retrieval	122
Reports not retrieved	5
Reports assessed for eligibility	117
Full-text reports excluded	106
Reports included	11
Independent randomised trials represented	8

Supplementary Table S13B. Aggregate primary reasons for full-text exclusion

Primary exclusion reason	n
Not randomised controlled trials	24
Population not eligible	28
Intervention not eligible / independent CCT effect not identifiable	34
Eligible transfer outcome not reported or not extractable	20
Total excluded full-text reports	106

Full-text eligibility assessment was completed for all 117 retrieved reports, and 106 reports were excluded after application of the prespecified criteria. The retained screening audit provides verified aggregate primary reasons and selected report-level examples; however, a single complete citation-linked export for all excluded reports was not preserved. Tables S13B-S13C therefore present the available verified audit trail without retrospectively reassigning reasons.

Supplementary Table S13C. Selected examples of potentially relevant randomised studies assessed against the current eligibility framework

Supplementary Table S13C. Selected examples of eligibility decisions (continued)

Report	Salient feature	Reason for different eligibility under the present review
Frain & Chen 2018	HIV-associated cognitive impairment/MCI population	Disease-dominant mixed populations were not included when a general-MCI subgroup was not separately extractable.
Bernini 2019 / Bernini 2021	Parkinson disease with MCI	PD-MCI was treated as a disease-dominant population outside the general MCI estimand.
Yeh 2022	Post-stroke MCI	Stroke-related MCI was treated as a distinct disease-dominant population.
Graessel 2024	Individualised CCT vs basic CCT	Pure CCT-versus-CCT head-to-head comparisons without an eligible non-CCT comparator did not contribute to the primary efficacy models.
Ferizaj 2025	Mean ages approximately 58-60 years in the two arms	The population did not clearly satisfy the age boundary applied to the middle-aged/older MCI target population.

These examples illustrate why the number of eligible trials may differ across reviews with broader population, comparator, and outcome definitions. They do not replace the aggregate PRISMA accounting in Table S13A-B.

Supplementary Appendix S4. Transfer-classification coding manual and decision rules

Purpose. The taxonomy separates direct task learning from cognitive near transfer, cognitive far transfer, and functional far transfer. Durability is treated as a time attribute rather than a separate construct. Classification is based on the relationship between training content and the independent outcome measure, not on the magnitude or statistical significance of the effect.

1. Identify trained domains. Use intervention manuals, protocols, task descriptions, and module lists to identify explicitly trained cognitive domains.

2. Identify the outcome domain. Map each outcome using the instrument's intended construct, the report authors' description, and conventional neuropsychological classification.

3. Direct task learning. Training-platform scores or substantively equivalent trained tasks are classified as direct task learning and excluded from the primary transfer models.

4. Cognitive near transfer. An independent outcome test is near transfer when its principal domain matches at least one cognitive domain explicitly trained by the intervention.

5. Cognitive far transfer. Global cognition or an independent test of an untrained cognitive domain is classified as cognitive far transfer.

6. Functional far transfer. ADL/IADL, everyday cognition, and performance-based real-world tasks are classified as functional far transfer.

7. Multidomain CCT. Independent tests of explicitly trained domains are near transfer; untrained-domain tests and global cognition are cognitive far transfer.

8. Time mapping. Each eligible outcome is mapped as immediate (<=4 weeks), early maintenance (>4 to <12 weeks), or sustained (>=12 weeks after cessation).

9. Effect visibility. Classification is performed without reference to the effect estimate, confidence interval, P value, or direction of benefit.

10. Adjudication. Two reviewers classify eligible outcomes independently; disagreements are resolved by discussion or third-reviewer adjudication using the same rules.

Supplementary Appendix S4. Transfer-classification coding manual and decision rules (continued)

Appendix S4, Table A. Construct definitions

Code	Construct	Operational definition	Primary use
direct	Direct task learning	Training-platform score or substantively equivalent trained task.	Supplementary/narrative only
near	Cognitive near transfer	Independent test in an explicitly trained cognitive domain.	M1 immediate; M3 sustained
cognitive_far	Cognitive far transfer	Global cognition or independent test in an untrained cognitive domain.	M2 immediate; M4 sustained
functional_far	Functional far transfer	ADL/IADL, everyday cognition, or performance-based everyday function.	M5 immediate; M6 sustained

Appendix S4, Table B. Classification decisions not permitted

Not permitted	Reason
Treating training-platform performance as near transfer	Would conflate task learning with transfer to an independent measure.
Pooling near, cognitive far, and functional far transfer into one overall effect	Would combine clinically and theoretically distinct estimands.
Pooling immediate and sustained outcomes in the same primary model	Would obscure durability and violate the defined time estimands.
Assigning transfer category solely from the test name	The same test can map differently when training content differs.
Using depression, anxiety, quality of life, or neuroimaging as substitutes for cognitive transfer	These are separate constructs.
Changing the selected outcome or category because an effect is statistically significant	Would create result-driven outcome selection.

Inter-rater reliability. The original pre-consensus independent coding sheets were not retained in a form that permits valid retrospective reconstruction of an agreement statistic. Cohen's kappa was therefore not calculated from the final consensus dataset. This limitation is reported explicitly in the manuscript.

Supplementary Table S16. Influence and outlier diagnostics

Supplementary Table S16. Influence diagnostics for pooled models M1-M5 (part 1 of 2)

Model	Study	k	Trial g	Variance	Full pooled g	Studentized deleted residual	Hat value	Cook distance (approx.)	DFBETAS	Pooled g if omitted	tau2 if omitted
M1	Fiatarone Singh	6	0.050	0.0149	0.139	-0.306	0.210	0.028	-0.235	0.172	0.112
M1	Duff	6	-0.250	0.0103	0.139	-1.902	0.221	0.604	-0.706	0.239	0.039
M1	Kua	6	0.510	0.0672	0.139	1.068	0.136	0.170	0.426	0.078	0.074
M1	Manenti	6	-0.110	0.0648	0.139	-0.681	0.138	0.079	-0.331	0.186	0.097
M1	Wu	6	0.580	0.0476	0.139	1.583	0.157	0.334	0.680	0.042	0.050
M1	Givon Schaham	6	0.280	0.0663	0.139	0.360	0.137	0.025	0.118	0.122	0.099
M2	Fiatarone Singh	8	-0.070	0.0396	0.230	-0.465	0.130	0.037	-0.214	0.280	0.452
M2	Duff	8	-0.200	0.0143	0.230	-0.700	0.139	0.088	-0.309	0.302	0.430
M2	Dowell-Esquivel	8	-0.350	0.0442	0.230	-0.938	0.129	0.135	-0.372	0.317	0.396
M2	Kua	8	0.110	0.1214	0.230	-0.174	0.109	0.004	-0.087	0.250	0.457
M2	Manenti	8	0.110	0.0798	0.230	-0.181	0.119	0.005	-0.095	0.252	0.463
M2	Wu	8	1.970	0.1206	0.230	4.847	0.109	0.834	0.981	0.000	0.034
M2	Givon Schaham	8	0.150	0.0332	0.230	-0.129	0.132	0.003	-0.079	0.248	0.472
M2	Montero-Odasso	8	0.390	0.0345	0.230	0.236	0.132	0.011	0.078	0.212	0.464
M3	Fiatarone Singh	4	-0.010	0.0149	-0.065	0.578	0.386	0.210	0.458	-0.100	0.000
M3	Duff	4	-0.120	0.0131	-0.065	-0.638	0.439	0.319	-0.564	-0.022	0.000
M3	Manenti	4	-0.160	0.0656	-0.065	-0.387	0.088	0.014	-0.120	-0.056	0.000
M3	Givon Schaham	4	0.060	0.0656	-0.065	0.512	0.088	0.025	0.159	-0.077	0.000
M4	Fiatarone Singh	5	-0.180	0.0396	0.017	-0.854	0.160	0.145	-0.384	0.054	0.023
M4	Duff	5	-0.190	0.0166	0.017	-1.980	0.258	0.541	-0.942	0.109	0.000
M4	Manenti	5	0.020	0.0795	0.017	0.014	0.096	0.000	0.014	0.015	0.027
M4	Givon Schaham	5	0.260	0.0334	0.017	1.164	0.178	0.289	0.533	-0.036	0.020

Supplementary Table S16. Influence diagnostics for pooled models M1-M5 (part 2 of 2)

Model	Study	k	Trial g	Variance	Full pooled g	Studentized deleted residual	Hat value	Cook distance (approx.)	DFBETAS	Pooled g if omitted	tau2 if omitted
M4	Montero-Odasso	5	0.150	0.0106	0.017	0.899	0.308	0.369	0.604	-0.043	0.021
M5	Fiatarone Singh	3	-0.310	0.0406	-0.160	-0.909	0.326	0.400	-0.632	-0.087	0.000
M5	Duff	3	-0.150	0.0358	-0.160	0.060	0.370	0.002	0.046	-0.165	0.003
M5	Dowell-Esquivel	3	-0.010	0.0435	-0.160	0.860	0.304	0.323	0.569	-0.225	0.000

Diagnostic interpretation. Wu et al. (2023) was the dominant influential observation in M2: its deletion reduced the pooled estimate from approximately g=0.23 to g=0.00 and the estimated between-trial variance from approximately tau2=0.38 to approximately 0.03. This diagnostic does not justify post hoc removal; it demonstrates the dependence of the primary M2 estimate on one influential high-risk-of-bias trial.

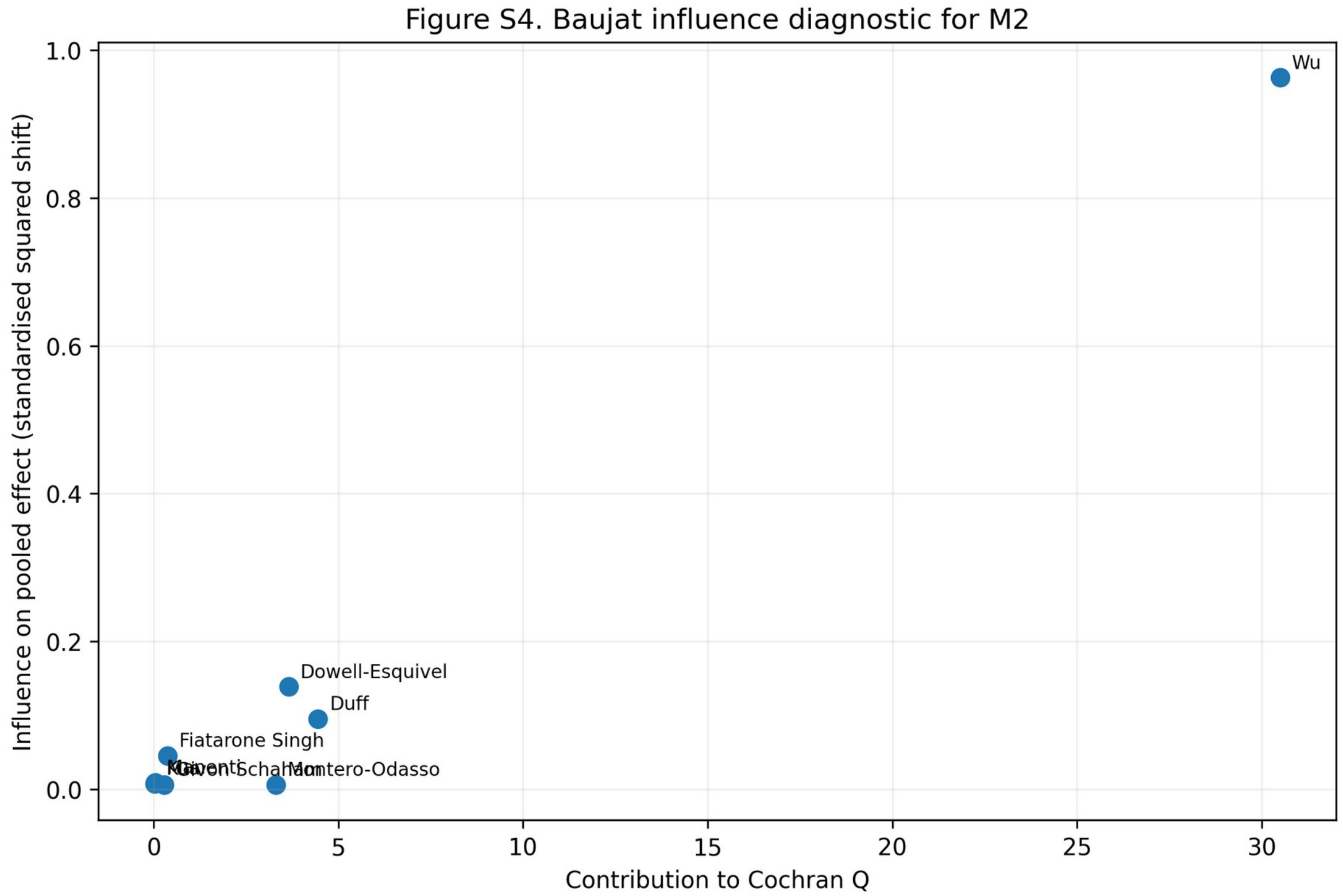

Figure S4. Baujat-style influence plot for M2 (immediate cognitive far transfer). The horizontal axis shows each trial's contribution to Cochran's Q; the vertical axis shows the standardised squared shift in the pooled effect after deletion. Wu et al. (2023) dominates both heterogeneity contribution and pooled-effect influence.

Supplementary Table S18. Long-term cognitive worsening and conversion-to-dementia information

Trial	Longest relevant follow-up	Conversion/progression information	Interpretation
SMART	18 months (approximately 12 months after cessation)	No adjudicated conversion-to-dementia endpoint identified in the retained reports.	Cognitive and ADL outcomes were reported; these cannot be reinterpreted as dementia conversion.
Duff 2022	1 year	No formal conversion endpoint. The report noted six participants with functional difficulties suggestive of dementia, equally represented across experimental and control groups; chi-square(1)=0.43, p=0.41.	The authors explicitly noted that one year was not sufficient to examine progression to dementia; no causal inference on conversion is possible.
Givon Schaham 2024	6 months	No adjudicated conversion-to-dementia endpoint identified.	Cognitive follow-up only; substantial attrition limits inference.
Dowell-Esquivel 2024 / Harvey 2025	30 days after cessation	No conversion-to-dementia endpoint.	Follow-up too short for a progression analysis.
Kua 2025	End of 10-week intervention	No conversion-to-dementia endpoint.	Pilot trial; no long-term progression follow-up.
Manenti 2020	Approximately 7 months from baseline	No adjudicated conversion-to-dementia endpoint identified.	Cognitive and ADL/IADL outcomes were reported.
SYNERGIC	Up to 12 months	No adjudicated conversion-to-dementia endpoint identified in the retained primary/secondary reports used here.	Cognitive and functional outcomes cannot be substituted for a conversion endpoint.
Wu 2023	Within approximately 1 week after 8-week intervention	No conversion-to-dementia endpoint.	No long-term clinical progression follow-up.

No valid quantitative synthesis of conversion to dementia was possible because adjudicated conversion was not a prespecified, consistently reported outcome across the included trials. A stable or near-null group difference in a cognitive score at follow-up should not be interpreted as evidence that CCT prevents, accelerates, or has no effect on clinical conversion to dementia.

Supplementary Appendix S5. PROSPERO registration information and deviations

The review was registered in PROSPERO as CRD420261463612. The registration record was checked against the final review methods and is summarised below.

Registration element	Recorded information
PROSPERO ID	CRD420261463612
First submitted to PROSPERO	29 July 2026
Date registered	29 July 2026
Review start date	28 July 2026
Estimated completion date	31 December 2026
Registered title/objective	Computerised cognitive training in older adults with MCI; cognitive transfer and durability.
Population	Clinically diagnosed MCI; general-MCI population rather than dementia or screening-only cognitive impairment.
Intervention	Structured computerised cognitive training delivered through digital platforms.
Comparator	Usual care and active controls.
Study design	Randomised controlled trials.
Risk-of-bias / certainty tools	Cochrane RoB 2 and GRADE.
Review stage at registration	Pilot work and formal searching/study identification had started; screening, data extraction, risk-of-bias assessment, and synthesis were not marked as started.

Supplementary Appendix S5. PROSPERO registration information and deviations (continued)

The exported PROSPERO record contained several fields that did not correspond to the review title and objectives. These discrepancies are reported transparently below and were not treated as prespecified transfer outcomes.

Field	PROSPERO record	Final review handling
Main outcomes / planned synthesis	The exported record contains fall-related psychological outcomes (balance confidence, concern about falling, traditional falls efficacy) and exercise-group terminology.	These fields are inconsistent with the registered CCT/MCI transfer title and objectives and were not used as the transfer outcomes for the present review.
Information sources	CINAHL, Embase, MEDLINE, PubMed, and Scopus were listed.	The final search additionally included Web of Science Core Collection, the Cochrane Library, and Wiley Online Library; all seven final search strategies are reported in Table S1.
Language restriction	No language restriction was recorded.	The final review included formally published English-language reports only; this deviation is acknowledged as a limitation.
Transfer framework / time windows	The exported outcome fields do not specify the final near/far/functional transfer models or immediate/sustained windows.	The final transfer framework, time windows, aggregation, and sensitivity rules are documented in the Methods, Tables S4-S6 and S12, and Appendix S4.
Complete protocol availability	The record states that a complete protocol had been written but was not publicly deposited.	The review reports the available registration information and methodological documentation without implying that the inconsistent outcome fields prespecified the final transfer analyses.
Funding	No specific/external funding; institutional support was indicated in the registration record.	No grant or external financial support was received; the institutional entry in PROSPERO reflects author affiliation rather than project funding.

The complete PROSPERO record is provided as a separate supplementary file.
